# Robust Huber-LASSO for improved prediction of protein, metabolite and gene expression levels relying on individual genotype data

**DOI:** 10.1093/bib/bbaa230

**Published:** 2020-10-16

**Authors:** Heike Deutelmoser, Dominique Scherer, Hermann Brenner, Melanie Waldenberger, INTERVAL study, Karsten Suhre, Gabi Kastenmüller, Justo Lorenzo Bermejo

**Affiliations:** Statistical Genetics Research Group, Institute of Medical Biometry and Informatics, Heidelberg University, Germany; Statistical Genetics Research Group, Institute of Medical Biometry and Informatics, Heidelberg University, Germany; Division of Preventive Oncology and the Division of Clinical Epidemiology and Aging Research at the German Cancer Research Center, Heidelberg, Germany; Research Unit Molecular Epidemiology and Institute of Epidemiology, Helmholtz Center Munich, Germany; University of Cambridge, Cambridge, UK; Weill Cornell Medicine and the Director of the Bioinformatics and Virtual Metabolomics Core at the Cornell campus in Doha, Qatar; Institute of Computational Biology, Helmholtz Center Munich, Germany; Statistical Genetics Research Group at the Institute of Medical Biometry and Informatics, Heidelberg University, Germany

**Keywords:** LASSO, robust statistics, Huber loss function, genetic prediction, molecular data

## Abstract

Least absolute shrinkage and selection operator (LASSO) regression is often applied to select the most promising set of single nucleotide polymorphisms (SNPs) associated with a molecular phenotype of interest. While the penalization parameter *λ* restricts the number of selected SNPs and the potential model overfitting, the least-squares loss function of standard LASSO regression translates into a strong dependence of statistical results on a small number of individuals with phenotypes or genotypes divergent from the majority of the study population—typically comprised of outliers and high-leverage observations.

Robust methods have been developed to constrain the influence of divergent observations and generate statistical results that apply to the bulk of study data, but they have rarely been applied to genetic association studies. In this article, we review, for newcomers to the field of robust statistics, a novel version of standard LASSO that utilizes the Huber loss function. We conduct comprehensive simulations and analyze real protein, metabolite, mRNA expression and genotype data to compare the stability of penalization, the cross-iteration concordance of the model, the false-positive and true-positive rates and the prediction accuracy of standard and robust Huber-LASSO.

Although the two methods showed controlled false-positive rates ≤2.1% and similar true-positive rates, robust Huber-LASSO outperformed standard LASSO in the accuracy of predicted protein, metabolite and gene expression levels using individual SNP data. The conducted simulations and real-data analyses show that robust Huber-LASSO represents a valuable alternative to standard LASSO in genetic studies of molecular phenotypes.

## Introduction

### Regularized regression models

Regularized regression is often applied in genetic studies of molecular phenotypes to select the most promising set of variants associated with a phenotype of interest. Several methods for regularized regression have been proposed. The basic idea behind regularization consists of adding a penalty term to the least-squares loss function—the sum of squared differences between the actual value of the response variable and the value predicted by the regression model—which is minimized to find the parameter values for the model that fits best to the data. A widely applied regularized regression method is the least absolute shrinkage and selection operator (LASSO), which adds a penalty term for the shrinkage of the parameter estimates to the least-squares loss function [1]

$$ \underset{\beta }{\min}\frac{1}{n}{\sum}_{i=1}^n{\left({y}_i-{\sum}_{j=1}^p{\beta}_j{x}_{ij}\right)}^2+\lambda{\sum}_{j=1}^p\left|{\beta}_j\right|. $$

In genetic studies of molecular phenotypes, the response variable *y_i_* represents the level of a particular compound measured in biological samples of individual *i*, for example the level of a given protein measured in urine, the concentration of a specific metabolite determined in plasma or the abundance of a particular mRNA in a particular tissue. *n* represents the number of individuals; *β_j_* is the regression coefficient for a predictor variable *j*, e.g. the age, gender or genotype of an individual for a particular single-nucleotide polymorphism (SNP). *x_ij_* represents the value taken by the predictor variable *j* in individual *i*. The penalty term consists of the regularization parameter *λ* multiplied by the sum of the absolute values of the regression coefficients—the so-called L1 norm. The parameter *λ* controls the extent of shrinkage of the parameter estimates: the higher the value of *λ*, the stronger the penalty and the closer to zero the parameter estimates. This penalization leads to a selection of variables, since the regression coefficients of explanatory variables with a low effect on the response variable *y_i_* are set to zero. Thus, the fitted regression model does not include all the predictors in the original dataset but only those variables with the strongest effect on the molecular phenotype, circumventing the necessity for explicit multiple testing correction; this leads more interpretable prediction models and also prevents overfitting of the model. The use of LASSO is increasingly common in the field of genetic association studies [2–5].

### Robust version of standard LASSO

Regression models that rely on the minimization of a least-squares loss function are considerably influenced by a small number of observations departing from the bulk of study data [6–8]. In the context of genetic studies of molecular phenotypes, these divergent observations may include both individuals with a response variable *y_i_* that does not follow the general trend of the study population—i.e. outliers—and also individuals with extreme values for single predictors (e.g. the individual age) or unusual predictor combinations (e.g. divergent multi-SNP genotypes)—so-called high-leverage observations.

The identification of divergent observations is particularly challenging in high-dimensional molecular studies, and even if individuals with atypical phenotypes and/or genotypes can be identified, the definition of criteria for their exclusion is always arbitrary. A much more promising unbiased and efficient approach relies on constraining the influence of outlying and high-leverage observations by means of robust statistical methods. Robust statistics aim to infer the best prediction model for the majority of the study population instead of the best model for any observation.

In this study, as an alternative to the least-squares loss function of standard LASSO, we consider the Huber loss function introduced by Rosset and Zhu [9]

$$ \underset{\beta }{\min}\frac{1}{n}{\sum}_{i=1}^n\rho \left({y}_i-{\sum}_{j=1}^p{\beta}_j{x}_{ij}\right)+\lambda{\sum}_{j=1}^p\mid{\beta}_j\mid, $$

where *ρ* represents the following function:

$$ {\rho}_{Huber}\left({e}_i\right)=\left\{\kern-6pt\begin{array}{c}\frac{1}{2}{e}_i^2\kern4.5em if\ \left|{e}_i\right|\le c\\{}c\ \left|{e}_i\right|-\frac{1}{2}{c}^2\kern1.5em \mathrm{otherwise}\end{array}\right. $$

with tuning constant $0<c\in \mathbb{R}$ and residual *e_i_*:

$$ {e}_i={y}_i-{\sum}_{j=1}^p{\beta}_j{x}_{ij}. $$

The tuning constant *c* regulates the influence of divergent observations on the prediction model, with smaller *c* values translating into more robust results. In this study, we choose in the first instance a *c* value equal to 1.345 to ensure 95% efficiency when the residuals are approximately normally distributed [10].

The derivative of the loss function *ρ* is called the influence curve and is represented by *Ψ*, and the weight function *w* is defined by

$$ w\left({e}_i\right)=\psi \left({e}_i\right)/{e}_i $$

translating into the following expressions:

$$ {\psi}_{\mathrm{Least}-\mathrm{squares}}\left({e}_i\right)={e}_i\kern3.5em {w}_{\mathrm{Least}-\mathrm{squares}}\left({e}_i\right)=1 $$

$$\begin{array}{l} {\psi}_{\mathrm{Huber}}\left({e}_i\right)=\left\{\kern-6pt\begin{array}{c}\ {e}_i\kern4.5em \mathrm{if}\ \left|{e}_i\right|\le c\\{}\ c \operatorname{sign}\left({e}_i\right)\kern1em \mathrm{otherwise}\end{array}\kern2.5em \right.\\[10pt] {w}_{\mathrm{Huber}}\left({e}_i\right)=\left\{\kern-5pt\begin{array}{c}\ 1\kern4.5em \mathrm{if}\ \left|{e}_i\right|\le c\\{}c/\left|{e}_i\right|\kern3em \mathrm{otherwise}\end{array}\right.\kern-5pt.\end{array} $$

The weight functions can be interpreted as follows: least-squares regression assigns the same weight to all observations, while the more robust Huber regression assigns lower weights to observations with large residuals. The algorithm for the computation of the robust LASSO estimators utilizing the Huber loss function was developed by Yi and Huang [11]. For a derivation of the influence curve taking into account the penalty term of the robust Huber-LASSO, see for example [12].

In this article, we explore the potential advantage of a robust version of standard LASSO that primarily utilizes the Huber loss function—hereinafter, ‘robust Huber-LASSO’—in the context of genetic studies of molecular phenotypes. We use real protein, metabolite, gene expression, and SNP data to design a comprehensive set of simulations under plausible study scenarios and compare the results from standard LASSO and robust Huber-LASSO based on simulated and real data.

## Materials and methods

### Data simulation

#### Study population

For the simulations, we used real genotype and protein data from 3301 individuals, who were randomly selected from the INTERVAL study, a genomic bioresource of about 50 000 generally healthy volunteers recruited between 2012 and 2014 at 25 centers of the UK’s NHS Blood and Transplant [data available through the European Genotype Archive (EGA), accession number: EGAS00001002555] [13, 14]. Study participants were genotyped using the Affymetrix Axiom UK Biobank genotyping array, which includes approximately 830 000 SNP positions. After quality control of samples and variants, genotypes were phased using SHAPEIT3 and imputed using a combined 1000 Genomes Phase 3-UK10K reference panel, resulting in 87 696 888 imputed variants in total [14]. An aptamer-based multiplex protein assay (SOMAscan) was used to measure the levels of 3622 proteins in plasma in two subcohorts of 2481 and 820 individuals each. Relative protein abundances were first natural log-transformed within each subcohort and then adjusted in a linear regression for age, sex, time from blood draw to processing (binary, ≤1 day/>1 day), and the first three principal components of ancestry from multi-dimensional scaling.

We used the residuals from the adjusted linear regression model as the response variable in our simulations. As potential predictors, we included the genotypes of the SNPs associated with the response variable in the INTERVAL study together with 1000 randomly selected, non-associated variants (reported as non-significantly associated with a probability value (*P*) ≥ 1.5 × 10^−11^) [14].

#### Simulation of outliers and selection of low-leverage and high-leverage individuals

We simulated outlying protein levels for individuals with average genotypes (low-leverage observations) and for individuals with divergent genotypes (high-leverage points). As shown with more detail in the Results section, the number of association signals for each protein in the INTERVAL study ranged from one (1206 proteins) to five (1 protein) [14]. In order to compare standard and robust Huber-LASSO, we selected proteins with three association signals (47 proteins) and calculated their proportions of explained variance using the following formula:

$$ \mathrm{Explained}\ \mathrm{variance}={\sum}_{k=1}^m2\cdot{\mathrm{MAF}}_k\left(1-{\mathrm{MAF}}_k\right){a}_k, $$

where *m* represents the number of association signals (*m* = 3 for our protein selection), MAF*_k_* is the minor allele frequency for each lead SNP and *a_k_* is the effect size for each lead SNP reported in the INTERVAL study [14, 15]. The median proportion of explained variance for the proteins with three associated SNPs in the INTERVAL study was 0.10, and the three SNPs associated with the protein beta defensin 119 (DEFB119, aptamer DEFB119.13455.10.3), taken together, also explained 0.10 of the variance, motivating us to use the residuals of the linear model for the protein DEFB119 as the response variable in our simulations.

Individuals with low- and high-leverage genotypes were selected based on the trivariate depth from a genetic principal component analysis (PCA) of the study population, which was conducted using the *eigenstrat* function available at www.popgen.dk/software/index.php/Rscripts. The trivariate depth of an observation relative to a finite set in the three-dimensional space is defined as the smallest number of observations lying in any closed half-space determined by a hyperplane through this observation [16]. The trivariate depth of each individual genotype was calculated using the R-package ‘depth’ [17].

#### Comparison of standard and robust Huber-LASSO models

**Figure 1:**
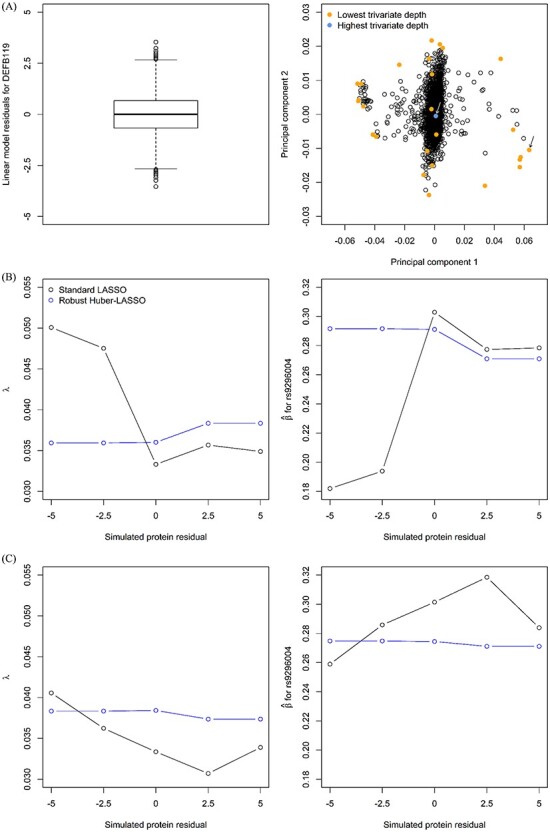
(**A**) Boxplot of the residuals from the linear model for the protein DEFB119 (left panel) and results from a genetic PCA for 3301 individuals from the INTERVAL study (right panel). (**B**) Dependence of the regularization parameter *λ* on the value assigned to the residual for the individual with the average genotype (left panel); dependence of the estimated regression coefficient for the SNP most strongly associated with the plasma level of protein DEFB119 (rs9296004) on the value assigned to the residual for the individual with the average genotype (right panel). (**C**) Dependence of the regularization parameter *λ* on the value assigned to the residual for the individual with the divergent genotype (left panel); dependence of the estimated regression coefficient for the SNP most strongly associated with the plasma level of protein DEFB119 (rs9296004) on the value assigned to the residual for the individual with the divergent genotype (right panel).

For each of 100 iterations, we randomly chose 500 of the 3301 INTERVAL participants and identified the two of these 500 individuals with the lowest and highest trivariate depth, representing a high-leverage and a low-leverage genotype, respectively (see the right panel of Figure 1A for an intuitive interpretation). We assigned an artificial residual that ranged from −5 to 5 to the individual with the low-leverage (scenario 1) or high-leverage (scenario 2) genotype, fitted the standard and robust Huber-LASSO models and predicted the residual values for the protein DEFB119 based on the fitted regression model and the individual genotypes. The R package ‘hqreg’ was used to fit the standard and robust Huber-LASSO models using an optimal *λ* obtained by tenfold cross-validation [11].

We compared standard LASSO and robust Huber-LASSO regarding the stability of the penalization parameter, the cross-iteration concordance of the model, the false-positive rate, the true-positive rate and the accuracy of predicted protein residuals. The stability of the penalization parameter assessed the impact of divergent observations on the regularization parameter *λ*. The cross-iteration concordance of the model was quantified by the average Jaccard similarity coefficient between the [100 × 99/2] = 4950 pairs of fitted models, calculated as follows:

$$ \mathrm{Jaccard}\ \mathrm{in}\mathrm{dex}=\frac{\mathrm{Number}\ \mathrm{of}\ \mathrm{SNPs}\ \mathrm{shared}\ \mathrm{by}\ \mathrm{the}\ \mathrm{two}\ \mathrm{models}}{\mathrm{Number}\ \mathrm{of}\ \mathrm{SNPs}\ \mathrm{retained}\ \mathrm{in}\ \mathrm{at}\ \mathrm{least}\ \mathrm{one}\ \mathrm{of}\ \mathrm{the}\ \mathrm{two}\ \mathrm{models}} $$

A Jaccard index equal to 1 implies identical predictor SNPs in the two models, and a Jaccard index of 0 means that the two models share no single predictor. The false-positive rate was estimated as the average over 100 iterations of the number of selected non-associated SNPs divided by the total number of non-associated variants (we randomly selected 1000 variants not associated with the protein residuals). The true-positive rate was calculated as the number of times an associated SNP was selected divided by the number of iterations. Confidence intervals for the estimated regression coefficients were computed based on the empirical distribution of the estimates over 100 iterations. The accuracy of the predicted protein residuals was assessed by 5-fold cross-validation: out-of-sample predictions were obtained for each individual, and then, the square of the Fisher-consistent version of Kendall’s correlation between observed and out-of-sample predicted values was computed as robust measure of the prediction accuracy [18].

We also examined the potential effects of (i) other values of the tuning constant for the Huber loss function (*c* equal to 1.20 and 1.80, which result in 90 and 99% efficiency under normality, respectively), (ii) the quantile loss function implemented in the R package ‘hqreg’ as an alternative to the Huber loss function, and (iii) a larger proportion of outliers (5/500 = 1% of the study population) instead of only one single outlier.

### Real-data applications

#### KORA

In addition to the above-described simulations based on real protein plasma data, we also compared standard and robust Huber-LASSO utilizing metabolite fasting serum data from the Cooperative Health Research in the Augsburg Region (*Kooperative Gesundheitsforschung in der Region Augsburg*, KORA) study. Out of the 4621 KORA S4 samples collected in the Augsburg region of Germany between 1999 and 2001 which were profiled using liquid-phase chromatography and gas chromatography separation coupled with tandem mass spectrometry, we analyzed 1408 samples with existing metabolite and genotype data. Association statistics between the SNPs and the serum metabolite levels were retrieved from a meta-analysis of KORA and TwinsUK data [19]. The median proportion of explained variance in the KORA study for metabolites with four associated SNPs was 0.10, and one metabolite showed associations with six SNPs (left panel of Figure 3A). As representatives for the comparison between standard and robust Huber-LASSO based on real metabolite data, we separately used the two metabolites L-carnitine (HMDB0000062, four associated SNPs, explained variance = 0.11) and glutarylcarnitine (HMDB0013130, six associated SNPs, explained variance = 0.14). For each metabolite the associated SNPs together with 100 randomly selected, non-associated variants (*P* ≥ 1.03 × 10^−10^ = 5 × 10^−8^/486 metabolites) were considered as potential predictors and metabolite levels were predicted using 5-fold cross-validation [19].

#### GTEx

In our second real-data application, we compared standard LASSO and robust Huber-LASSO based on mRNA expression and genotype data from 621 healthy donors provided by the Genotype-Tissue Expression (GTEx) project (https://gtexportal.org). We separately used the expression levels of the genes *AGA* (3 unlinked, associated SNPs, explained variance = 0.06), *SNRNP25* (5 unlinked, associated SNPs, explained variance = 0.069) and *XRRA1* (3 unlinked, associated SNPs, explained variance = 0.0004) as response variables to compare the standard and robust regularized regression methods under a wide range of different situations—e.g. a low explained variance for *XRRA1* (left panel of Figure 3B). PrediXcan is a popular software for the prediction of mRNA expression based on individual genotype data [20]. The SNPs associated with the expression of the corresponding genes according to PrediXcan (SNPs with estimated regression coefficients different from zero), together with 2000 randomly selected, non-associated variants (those with estimated regression coefficients equal to zero according to PrediXcan), were considered as potential predictors. In consistency with the analyses described above, we applied 5-fold cross-validation to predict the expression levels using standard and robust Huber-LASSO and compared the prediction accuracies relying on the square of the Fisher-consistent version of Kendall’s correlation—hereinafter, ‘squared correlation’.

#### INTERVAL

The third real-data application considered 500 randomly selected individuals from the INTERVAL study [14]. The residuals from the linear models for the proteins DEFB119 (aptamer DEFB119.13455.10.3) and SLAM family member 7 (SLAMF7, aptamer SLAMF7.7882.31.3) were used as separate response variables, because the proportion of variance explained by their associated SNPs—0.10 and 0.11, respectively—was similar to the median proportion of explained variance (0.10) for proteins with three associated SNPs in the INTERVAL study (left panel of Figure 3C). Moreover, the genetic associations for SLAMF7 have been replicated in several studies [21]. The three associated SNPs for each protein, together with 1000 randomly selected, non-associated variants, were considered as potential predictors. Once again, we predicted the residuals for each protein based on the fitted models and the individual genotypes and quantified the prediction accuracy by the squared correlation between observed and predicted values, applying 5-fold cross-validation.

## Results

### Motivating example

Before presenting the simulation results, we illustrate the lack of robustness of standard LASSO against a single divergent observation with an example. Figure 1A shows a boxplot of the residuals from the linear model for the protein DEFB119 (left panel) and the results from a genetic PCA for 3301 individuals from the INTERVAL study (right panel). The residuals from the linear model for the protein DEFB119 varied from −3.5 to 3.5. The genotypes of the individual depicted in blue in the right panel of Figure 1A resulted in the highest trivariate depth (low-leverage genotype). The individuals depicted in orange showed the lowest trivariate depth, and the individual marked with an arrow was used in this motivating example as the high-leverage genotype.

To examine the influence of a single outlying observation on the results from standard and robust Huber-LASSO, we assigned residual values from −5 to 5 to the individual with the ‘average genotype’ (low-leverage genotype represented by a blue dot in Figure 1A). The left panel in Figure 1B shows the dependence of the regularization parameter *λ* (*y*-axis) on the value assigned to the residual of the individual with the average genotype (*x*-axis). The plot clearly shows that the penalization parameter was more stable for robust Huber-LASSO (blue line, range of *λ*: 0.036–0.038, monotonic trend) than for standard LASSO (black line, range of *λ*: 0.033–0.050, non-monotonic trend). The right panel in Figure 1B shows the dependence of the estimated regression coefficient for the SNP (rs9296004) most strongly associated with the plasma level of protein DEFB119 on the value assigned to the residual. The single outlying observation showed a greater influence on the standard (range of $\hat{\beta}:$0.18–0.30 and non-monotonic trend) than on the robust results (range of $\hat{\beta}:$0.27–0.29, monotonic trend).

We also examined the influence of a single outlying observation on the results from standard and robust Huber-LASSO when residuals from −5 to 5 were assigned to the individual with a ‘divergent genotype’ (the high-leverage genotype represented by the orange dot marked by an arrow in Figure 1A). The results are shown in Figure 1C. Again, a single outlying observation influenced the results from standard LASSO more strongly than those from robust Huber-LASSO. Interestingly, the single individual with the average genotype and the outlying phenotype exhibited a stronger impact on the results than the individual with the divergent genotype and the outlying phenotype, as reflected by the larger variability of *λ* and $\hat{\beta}$in Figure 1B than in Figure 1C. The ratios between the estimated regression coefficients for the three SNPs associated with the plasma level of protein DEFB119 are shown in Supplementary Figure 1.II, available online at https://academic.oup.com/bib. They confirm that results from standard LASSO can be heavily influenced by a small number of outliers: in this motivating example, the SNP rs12301299 was not included in the standard LASSO regression model when negative residuals were assigned to the individual with an average genotype (A). In general, the ratios between the regression coefficients tended to be more stable for robust Huber-LASSO than for standard LASSO. Supplementary Figure 1.I, available online at https://academic.oup.com/bib, compares the influence on *λ* and $\hat{\beta}$of a single outlying residual from −5 to 5 assigned to individuals with an average (A) or a divergent (B) genotype, considering also the robust quantile-LASSO.

This example demonstrates that results from standard LASSO can be heavily influenced by a small number of outlying observations and that results from the robust Huber-LASSO and quantile-LASSO tend to be more stable. In the following simulations, we compare the two methods more thoroughly.

### Simulation results

The median value of the regularization parameter over 100 iterations in the absence of outliers, i.e. considering the actual residuals for the protein DEFB119 for all individuals, was *λ* = 0.041 for standard and *λ* = 0.044 for robust Huber-LASSO (Table 1). The assignment of a single outlying residual equal to ±5 for individuals with an average genotype translated into an *λ* increase of 0.002–0.003 for standard LASSO compared with 0.000–0.002 for robust Huber-LASSO. The assignment of a residual equal to ±5 for individuals with a divergent genotype translated into an *λ* increase of 0.002–0.003 for standard LASSO, whereas *λ* did not change in robust Huber-LASSO.

**Table 1:** Regularization parameter *λ*, Jaccard index and false-positive rate from standard and robust Huber-LASSO

Simulated outlying phenotype	Genotype	Standard LASSO			Median λ	Robust Huber-LASSO	
		Median λ	Median Jaccard index	Median false-positive rate		Median Jaccard index	Median false-positive rate
None		0.041	0.125	0.020	0.044	0.111	0.018
5	Low-leverage	0.044	0.115	0.020	0.044	0.109	0.018
−5	(average)	0.043	0.125	0.019	0.046	0.111	0.016
5	High-leverage	0.044	0.119	0.020	0.044	0.111	0.019
−5	(divergent)	0.043	0.123	0.020	0.044	0.111	0.018

In the absence of outliers, the standard LASSO model showed a higher concordance across iterations (Jaccard index = 0.125) than robust Huber-LASSO (Jaccard index = 0.111); this difference was due to the stronger penalization and, subsequently, the lower number of variants selected by robust Huber-LASSO. The assignment of an outlying residual decreased more severely the cross-iteration concordance of the model for standard LASSO (max. reduction of the Jaccard index = 0.010) than for robust Huber-LASSO (max. reduction of the Jaccard index = 0.002).

The median false-positive rate over 100 iterations in the absence of outliers was 0.020 for standard LASSO and 0.018 for robust Huber-LASSO (Table 1). The inclusion of an artificial outlier influenced these rates only slightly (±0.002).

Supplementary Table 1.I, available online at https://academic.oup.com/bib, shows the corresponding results for other values of the tuning constant for the Huber loss function (*c* = 1.20, 1.80) and for the quantile loss function. A single outlying residual equal to ±5 for individuals with an average genotype translated into an *λ* increase of 0.000–0.001 for *c* = 1.20 compared with *λ* changes of −0.003 and 0.004 for *c* = 1.80. The assignment of a residual equal to ±5 for individuals with a divergent genotype did not influence *λ* for *c* = 1.20, whereas *λ* increased by 0.001 for *c* = 1.80. The median value of *λ* in the absence of outliers was *λ* = 0.022 for robust quantile-LASSO. The assignment of a residual equal to ±5 for individuals with a divergent genotype showed a larger influence on *λ,* and on the cross-iteration concordance of the model, for robust quantile-LASSO than for robust Huber-LASSO with *c* = 1.345. The false-positive rates for other values of the tuning constant of the Huber loss function and for the robust quantile-LASSO were ≤2.1%.

Supplementary Table 1.II, available online at https://academic.oup.com/bib, shows the respective results considering five outliers (1% of the study population). The increase in the proportion of outliers translated into larger *λ* increases for standard LASSO but did not affect *λ* for robust Huber-LASSO. It showed a stronger influence on the cross-iteration concordance of the model for standard LASSO than for robust Huber-LASSO, and false-positive rates remained ≤2%.

Table 2 shows the explained variance, the MAF and the reported effect size of the three SNPs associated with the plasma levels of protein DEFB119, as well as the corresponding true-positive rate and the estimated regression coefficients from standard and robust Huber-LASSO. In the absence of outliers, the true-positive rate for the SNPs rs9296004 and rs11845244 was identical with standard and robust Huber-LASSO; the true-positive rate for the SNP rs12301299 was higher for standard than for robust Huber-LASSO. The assignment of an outlying residual equal to ±5 generally showed a higher impact on the true-positive rate from standard than from robust Huber-LASSO.

**Table 2:** Explained variance, MAF and reported effect size of the three SNPs associated with the plasma levels of protein DEFB119, as well as the corresponding true-positive rate and the estimated regression coefficients from standard LASSO and robust Huber-LASSO

Associated SNP	Explained variance	MAF	Reported effect size	Standard LASSO				Robust Huber-LASSO			
				True-positive rate	Estimated coefficient			True-positive rate	Estimated coefficient		
					Mean	95% CI			Mean	95% CI	
No simulated outlier											
rs9296004	0.055	0.082	0.606	0.970	0.253	0.233	0.273	0.970	0.281	0.253	0.309
rs11845244	0.027	0.354	0.241	0.850	0.094	0.082	0.106	0.850	0.103	0.087	0.118
rs12301299	0.018	0.163	0.254	0.960	0.127	0.114	0.139	0.840	0.095	0.082	0.109
Average genotype											
Simulated protein residual = 5											
rs9296004	0.055	0.082	0.606	0.930	0.229	0.208	0.250	0.960	0.279	0.251	0.308
rs11845244	0.027	0.354	0.241	0.790	0.078	0.066	0.091	0.840	0.100	0.085	0.116
rs12301299	0.018	0.163	0.254	0.940	0.122	0.109	0.135	0.830	0.096	0.082	0.110
Simulated protein residual = −5											
rs9296004	0.055	0.082	0.606	0.950	0.247	0.226	0.269	0.950	0.287	0.258	0.317
rs11845244	0.027	0.354	0.241	0.860	0.095	0.082	0.108	0.860	0.107	0.091	0.123
rs12301299	0.018	0.163	0.254	0.960	0.121	0.108	0.134	0.870	0.096	0.083	0.109
Divergent genotype											
Simulated protein residual = 5											
rs9296004	0.055	0.082	0.606	0.960	0.230	0.21	0.251	0.960	0.281	0.253	0.310
rs11845244	0.027	0.354	0.241	0.810	0.090	0.078	0.103	0.840	0.104	0.088	0.120
rs12301299	0.018	0.163	0.254	0.950	0.120	0.107	0.133	0.860	0.097	0.084	0.111
Simulated protein residual = −5											
rs9296004	0.055	0.082	0.606	0.960	0.247	0.225	0.269	0.950	0.288	0.259	0.316
rs11845244	0.027	0.354	0.241	0.800	0.082	0.070	0.095	0.850	0.103	0.088	0.119
rs12301299	0.018	0.163	0.254	0.970	0.126	0.113	0.138	0.880	0.097	0.084	0.110

The estimated regression coefficients for the SNPs rs9296004 and rs11845244 were similar based on standard and robust Huber-LASSO (overlapping 95% confidence intervals) when no artificial outlier was included. In contrast, and consistently with the true-positive rate, the estimated regression coefficient for the SNP rs12301299 was higher with standard LASSO than with robust Huber-LASSO. As with the true-positive rate, the assignment of an outlying residual generally showed a higher impact on the estimated coefficients from standard than from robust Huber-LASSO. Note that the effect sizes reported by Sun *et al.* were calculated separately for each SNP, and the coefficients shown in Table 2 were estimated considering multiple variants simultaneously. Supplementary Table 2.I, available online at https://academic.oup.com/bib, shows the corresponding results for alternative values of the tuning constant of the Huber loss function (*c* = 1.20, 1.80) and for the quantile loss function, and Supplementary Table 2.II, available online at https://academic.oup.com/bib, shows the results for an outlier proportion equal to 1%. True-positive rates were quite close, with the overall ranking: standard LASSO > Huber-LASSO (*c* = 1.80) > Huber-LASSO (*c* = 1.345) > Huber-LASSO (*c* = 1.20) > quantile-LASSO. The average true-positive rates for the standard LASSO were 0.93 (no outlier), 0.91 (one outlier) and 0.81 (five outliers), compared with 0.89 (no outlier), 0.89 (one outlier) and 0.87 (five outliers) for the robust Huber-LASSO.

**Figure 2:**
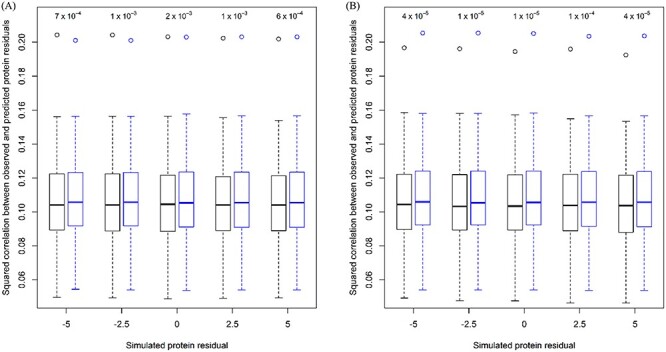
Boxplots of the squared correlation between the observed and the predicted DEFB119 protein residuals based on standard LASSO (black) and robust Huber-LASSO (blue) with simulated protein residuals for individuals with average (**A**) or divergent (**B**) genotypes. Probability values from two-sided paired *t*-tests are shown in the upper part of each panel.

The goal of genetic studies on molecular phenotypes often consists of predicting the phenotype as accurately as possible relying on individual genotype data. Figure 2 depicts the boxplots of the squared correlation between the observed and the predicted protein residuals based on standard LASSO (black) and robust Huber-LASSO (blue). With and without (simulated protein residual = 0) outliers for individuals with average (A) or divergent (B) genotypes, robust Huber-LASSO always resulted in a higher accuracy of the predicted DEFB119 residuals than standard LASSO (*P* from two-sided paired *t*-tests between 0.00001 and 0.002, Figure 2). More specifically, the median squared correlation varied from 0.104 to 0.105 for standard LASSO and was equal to 0.106 for robust Huber-LASSO. Supplementary Figures 2.I and 2.II, available online at https://academic.oup.com/bib, show the boxplots of the standard squared correlation for other values of the tuning constant of the Huber loss function, for the quantile loss function and for an outlier proportion equal to 1%. The median standard squared correlation amounted to 0.096 for robust Huber-LASSO with *c* = 1.80 and *c* = 1.20 and varied from 0.096 to 0.097 for robust quantile-LASSO. Compared with one single outlier, an outlier proportion equal to 1% hardly affected the median standard squared correlation. The use of the standard Pearson’s correlation, and the Fisher-consistent version of Spearman’s correlation [18], also corroborated the higher prediction accuracy of robust Huber-LASSO than standard LASSO (see Supplementary Figure 2.III available online at https://academic.oup.com/bib).

Recapitulating the simulation results, single outliers showed a greater influence on the regularization parameter, the cross-iteration concordance of the model, the true-positive rate and the estimated regression coefficients for standard LASSO than for robust Huber-LASSO. The investigated standard and robust regularized regression methods showed false-positive rates ≤2.1%. A higher prediction accuracy was noticed for robust Huber-LASSO than for standard LASSO.

### Real-data applications

**Figure 3:**
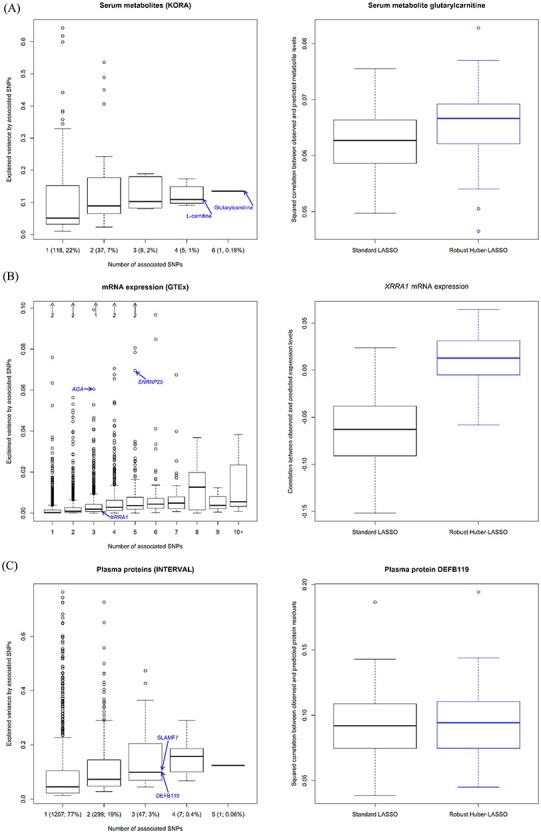
(**A**) Distribution of the explained variance for the serum metabolites analyzed in the KORA study with unlinked, associated SNPs (left panel); boxplots of the squared correlation from standard and robust Huber-LASSO for the metabolite glutarylcarnitine (right panel). (**B**) Distribution of the explained variance in mRNA expression for the genes investigated in the GTEx project with unlinked, associated SNPs according to PrediXcan (left panel). The black arrows show the number of genes with an explained variance >0.10; boxplots of the correlation coefficient from standard and robust Huber-LASSO for the gene *XRRA1* (right panel). (**C**) Distribution of the explained variance for the plasma proteins with unlinked, associated SNPs in the INTERVAL study (left panel); boxplots of the squared correlation from standard and robust Huber-LASSO for the protein DEFB119 (right panel). The blue arrows highlight the metabolites, genes and proteins selected for our real-data applications.

The left panel of Figure 3A shows the distribution of the explained variance for 169 serum metabolites analyzed in the KORA study with unlinked, associated SNPs. Note that about two-thirds (68%) of the 529 investigated metabolites showed no SNP association and are not represented in this figure. Among the investigated metabolites, 22% were associated with one SNP, 7% with two SNPs, 2% with three SNPs, 1% with four SNPs and the metabolite glutarylcarnitine with six SNPs. As expected, on average, the explained variance increased with the number of associated SNPs. The explained variance for the metabolites L-carnitine (0.11, associated with four SNPs) and glutarylcarnitine (0.14, associated with six SNPs), selected as representatives for our real-data applications, are indicated with blue arrows in the figure.

Table 3 shows the explained variance and the median squared correlation between observed and predicted levels of the metabolites L-carnitine and glutarylcarnitine, and the right panel of Figure 3A shows the boxplots of the squared correlation from standard and robust Huber-LASSO for the metabolite glutarylcarnitine. Robust Huber-LASSO resulted in better prediction accuracy than standard LASSO.

The left panel of Figure 3B shows the distribution of the explained variance in mRNA expression for 5612 genes investigated in the GTEx project with 1–15 unlinked, associated SNPs according to PrediXcan. The blue arrows highlight the three genes (*AGA*, *SNRNP25* and *XRRA1*) selected for our real-data applications. Table 3 shows the explained variance and the median squared correlation between observed and predicted expression levels for the three selected genes. Robust Huber-LASSO resulted in higher prediction accuracy than standard LASSO.

The ability to predict molecular phenotypes based on individual genotype data is limited by the explained variance [20]. Weak to null correlations between genetically observed and predicted expression levels are expected for genes with a small explained variance—a threshold value equal to 0.01 for the standard squared correlation has been proposed [22]. It is especially important for these genes to check the sign of the correlation coefficient. As an example, the explained variance in mRNA expression for *XRRA1* was 0.0004. The median correlation coefficient based on standard LASSO was −0.0629, compared with 0.0029 for robust Huber-LASSO. The right panel of Figure 3B shows the boxplots of the correlation coefficient from standard and robust Huber-LASSO for this gene.

Finally, the left panel of Figure 3C shows the distribution of the explained variance for 1561 plasma proteins measured in the INTERVAL study with unlinked, associated SNPs. The explained variance for the two representative proteins DEFB119, which was also used to design the conducted simulations, and SLAMF7 is indicated with blue arrows. The proteins were selected to represent the median explained variance by their number of associated SNPs. In agreement with the simulation results and with the analyses of real metabolite and gene expression data, robust Huber-LASSO consistently outperformed standard LASSO in prediction accuracy. Robust Huber-LASSO needed a longer computation time than standard LASSO. For illustration, the analysis of the real protein data took 0.7 min using one core for standard LASSO compared with 3.2 min for robust Huber-LASSO. Supplementary Table 3, available online at https://academic.oup.com/bib, shows the median squared correlation for other values of the tuning constant for the Huber loss function and for the quantile loss function. The different robust regularization methods always showed a higher prediction accuracy than standard LASSO, with small differences among the alternative robust techniques.

## Discussion

The aim of the present study was to investigate the benefits and limitations of a modified version of standard LASSO that uses the Huber loss function instead of the standard least-squares to cut down the influence of outlying molecular phenotypes, which can be found in individuals with average or divergent genotypes. Computer simulations were complemented with the analysis of three real molecular datasets to examine the stability of the regularization parameter *λ*, the cross-iteration concordance of the model, the false-positive rate, the true-positive rate and the correlation between predicted and observed levels of proteins, metabolites and gene expression using standard LASSO and robust Huber-LASSO.

**Table 3:** Explained variance and the median squared correlation between observed and predicted levels of the metabolites l-carnitine and glutarylcarnitine, the three genes *AGA*, *SNRNP25* and *XRRA1* and the proteins DEFB119 and SLAMF7

Outcome	Explained variance	Median squared correlation	
		Standard LASSO	Robust Huber-LASSO
Glutarylcarnitine metabolite level	0.135	0.063	0.067
l-Carnitine metabolite level	0.149	0.040	0.047
*AGA*expression level	0.060	0.048	0.052
*SNRNP25* expression level	0.069	0.045	0.054
*XRRA1* expression level	0.0004	0.004	0.0001
DEFB119 protein level	0.100	0.092	0.094
SLAMF7 protein level	0.108	0.104	0.107

False-positive rates were well controlled by the two methods. As expected, outlying phenotypes influenced the extent of penalization, the cross-iteration concordance of the model, the true-positive rate and the estimated regression coefficients less for robust Huber-LASSO than for standard LASSO. Interestingly, from the applied point of view, outlying phenotypes in individuals with average genotypes showed a greater impact on the results from regularized regression than outlying phenotypes in combination with divergent genotypes. Our main finding was that robust Huber-LASSO seems to outperform standard LASSO in the prediction of protein, metabolite and gene expression based on individual genotype data.

In the present study, we used the Fisher-consistent version of Kendall’s correlation between observed and genetically predicted protein, metabolite and gene expression measurements to quantify the prediction accuracy [18]. The classical Pearson’s estimator of correlation may be seriously affected by outliers, and robust correlation metrics such as the popular Kendall and Spearman correlations provide a good compromise between robustness and Gaussian efficiency. We used Kendall’s correlation in our study because it is more robust and slightly more efficient than the Spearman correlation [18]. The interpretation of the three correlation measures is similar, and Kendall’s, Spearman’s and Pearson’s correlation consistently pointed to a higher prediction accuracy of robust Huber-LASSO than standard LASSO in this study.

In our analyses of real mRNA expression data from the GTEx project, we applied 5-fold cross-validation: the standard and robust Huber-LASSO estimates of the regression coefficients were computed using four fifths of the data, and the gene expression levels were predicted in an independent dataset that contained the remaining one-fifth of the observations. PrediXcan, a popular software for the prediction of gene expression, applies a similar approach [20]. LASSO and Elastic Net were compared in the development of PrediXcan and showed similar performances, but the released version of PrediXcan utilizes Elastic Net. The potential improvement in the prediction accuracy of gene expression using an updated version of PrediXcan that incorporates the robust Elastic Net and/or LASSO is left for future research.

Another potential application of robust LASSO is Mendelian randomization (MR) [23]. MR uses genetic variants as instrumental variables to investigate the causal effect of an exposure on an outcome. The genetic associations must fulfil several assumptions for causality testing that are generally difficult to check. Causal effects are typically estimated by the inverse-variance weighted (IVW) method. Since the inclusion of just one invalid genetic variant in the MR analysis yields biased IVW estimators, the development of MR methods that are robust against invalid instruments is an active area of research. For example, Slob and Burgess recently compared MR-LASSO and a robust IVW version that uses Tukey’s biweight loss function (MR-Robust) [24]. Combination of the two methods (MR-Robust LASSO) could be advantageous and should also be investigated in the future.

A key limitation of genetic studies on molecular phenotypes is their dependence on strong associations between the genetic predictors and the outcome. It is important to note here that the identified prediction advantage of robust Huber-LASSO over standard LASSO was relatively small compared with this essential limitation. The explained variance is the upper limit of the correlation between predicted and observed phenotypes. The 0.01 cutoff for the squared correlation, previously applied to select phenotypes that can be genetically predicted, translates into a correlation coefficient between predicted and observed phenotypes >0.10 or <−0.10 [20, 22]. In general, the explained variance was smaller for mRNA expression than for plasma protein and serum metabolite levels. We found that robust Huber-LASSO improved the prediction accuracy for molecular phenotypes with a correlation coefficient >0.10; neither robust Huber-LASSO nor standard LASSO can accurately predict the outcome when the association between the genetic variants and the molecular phenotype is weak.

One of the strengths of our study was the investigation of the stability of the regularization parameter by means of a data-driven cross-validation approach instead of fixing the value of *λ* [12]. Outliers and high-leverage observations may also have an indirect effect on the estimated regression coefficients through the regularization parameter. Other novelties of this study were the investigation of the influence of outlying molecular phenotypes in combination with both average and divergent genotypes on the prediction results, and the examination of the number of associated genetic variants for three common molecular phenotypes. On the other hand, limitations of our study comprised the non-evaluation of alternative molecular markers, for example methylation and non-coding RNA expression, and the consideration of particular types of regularized regression and robust methods. There are regularized regression methods other than LASSO, such as Ridge regression and Elastic Net, which use distinct penalty terms [25–27]. The penalization of Ridge regression is based on the L2 norm, whereas Elastic Net combines the L1 and L2 norms in the penalty term. Robust versions of the Elastic Net using S- and MM-estimation have been proposed and are implemented in the R package pense [28]. Furthermore, instead of using the Huber loss function to limit the influence of outliers on a particular regularized regression method, other robust loss functions, such as the quantile, Hampel’s redescending, Tukey’s biweight and the least trimmed squares loss functions, could be used [29]. For example, a trimmed version of the LASSO is implemented in the R package robustHD. The present simulations, real datasets and results may guide the comparison of other regularized regression methods in conjunction with distinct robust loss functions in the context of genetic studies of molecular genotypes in the future.

### Data availability

The data underlying this article were kindly provided by EGA (https://www.ebi.ac.uk/ega/about/access, accession number: EGAS00001002555), KORA and the GTEx project (https://dbgap.ncbi.nlm.nih.gov, accession number: phs000424.v8.p2). Original data can be requested from the EGA or the GTEx project. The informed consents given by KORA study participants do not cover data posting in public databases. However, data are available upon request via the KORA Project Application Self-Service Tool (https://epi.helmholtz-muenchen.de/). Data requests can be submitted online and are subject to approval by the KORA board.

**Key Points**

- Robust Huber-LASSO outperformed standard LASSO in the prediction of protein, metabolite and mRNA expression levels based on individual genotype data.
- Single outliers exerted a greater influence on the regularization parameter, the cross-iteration concordance of the model, the true-positive rate and the estimated regression coefficients for the standard than for the robust Huber-LASSO.
- The two regularized regression methods showed well-controlled false-positive rates.

## Supplementary Material

SupplementaryMaterial_20200822_bbaa230

## Notes

**INTERVAL study**, University of Cambridge, Cambridge, UK.

## Notes

**Heike Deutelmoser** is a PhD student in the Statistical Genetics Research Group, Institute of Medical Biometry and Informatics, Heidelberg University, Germany, in cooperation with the Division of Preventive Oncology, German Cancer Research Center and National Center for Tumor Diseases Heidelberg, Germany, with a background in mathematics and statistical genetics. Her current research focuses on robust regularized regression in cancer prevention.

**Dominique Scherer** is a postdoc in the Statistical Genetics Research Group, Institute of Medical Biometry and Informatics, Heidelberg University, Germany, with a background in molecular genetic epidemiology. Her current research focuses on the use of genetic biomarkers for personalized cancer prevention and the effect of inflammation on cancer susceptibility and prognosis.

**Hermann Brenner** is a Professor of epidemiology and the Head of both the Division of Preventive Oncology and the Division of Clinical Epidemiology and Aging Research at the German Cancer Research Center, Heidelberg, Germany, with a background in medicine and epidemiology. His current research focuses on the prevention and early detection of cancer.

**Melanie Waldenberger** is the Head of the Complex Disease Group at the Research Unit Molecular Epidemiology and Institute of Epidemiology, Helmholtz Center Munich, Germany, with a background in biology and epidemiology. Her main research interest is the genetic, epigenetic and functional analysis of complex diseases, with a focus on cardiovascular and metabolic conditions and their interplay with environmental factors.

**INTERVAL study**, University of Cambridge, Cambridge, UK.

**Karsten Suhre** is a Professor of physiology and biophysics at Weill Cornell Medicine and the Director of the Bioinformatics and Virtual Metabolomics Core at the Cornell campus in Doha, Qatar. After a very interdisciplinary career in the theoretical physics, atmospheric sciences, bacterial and viral genomics, and protein bioinformatics, he is now investigating how human genetics, metabolomics and proteomics interact with environmental challenges and lifestyle factors in the development of complex disorders, especially diabetes, cancer, heart and kidney diseases.

**Gabi Kastenmüller** is the Head of the Systems Metabolomics Group at the Institute of Computational Biology, Helmholtz Center Munich, Germany, with a background in chemistry and bioinformatics. In her current research, she focuses on integrating metabolomics and other omics data into a systems medicine context to investigate how a person’s individual metabolic make-up, its changes over time and its link to genetic variation affect health and disease.

**Justo Lorenzo Bermejo** is a Professor of epidemiology and biostatistics and the Head of the Statistical Genetics Research Group at the Institute of Medical Biometry and Informatics, Heidelberg University, Germany, with a background in statistical genetics and genetic epidemiology. His current research focuses on the development and application of robust and Bayesian methods in statistical genetics and the identification of molecular markers for the personalized prevention and treatment of cancer.
