# Supplementary material for: Robust Huber-LASSO for improved prediction of protein, metabolite and gene expression levels relying on individual genotype data
Source: Brief Bioinform. 2020 Oct 16;22(4):bbaa230. doi: 10.1093/bib/bbaa230 (PMC8293825; doi:10.1093/bib/bbaa230)
Supplement: SupplementaryMaterial_20200822_bbaa230 [file supplementarymaterial_20200822_bbaa230.pdf]

Robust Huber-LASSO for improved prediction of protein, metabolite, and gene expression levels relying on individual genotype data

Author list: Heike Deutelmoser, Dominique Scherer, Hermann Brenner, Melanie Waldenberger, INTERVAL study, Karsten Suhre, Gabi Kastenmüller, Justo Lorenzo Bermejo

SUPPLEMENTARY DATA

Supplementary Figure 1.I (Extension of **Figure 1**): (**A**) Dependence of the regularization parameter λ (left panel) and of the estimated regression coefficient for the SNP most strongly associated with the plasma level of protein DEFB119 (right panel) on the value assigned to the residual for the individual with the average genotype considering the quantile loss function (**B**) Dependence of the regularization parameter λ (left panel) and dependence of the estimated regression coefficient for the SNP most strongly associated with the plasma level of protein DEFB119 (right panel) on the value assigned to the residual for the individual with the divergent genotype considering the quantile loss function.

Supplementary Figure 1.II (Extension of **Figure 1**): Dependence of the ratios between the estimated regression coefficients for the three SNPs most strongly associated with the plasma level of protein DEFB119 on the value assigned to the residual for the individual with average (**A**) and divergent (**B**) genotype.

Supplementary Figure 2.I (Extension of **Figure 2**): Boxplots of the standard squared correlation between the observed and the predicted DEFB119 protein residuals considering other values of the tuning constant for the Huber loss function (c = 1.20 and c = 1.80) and the quantile loss function with simulated protein residuals for individuals with average (**A**) or divergent (**B**) genotypes. From left to right: Standard LASSO (black); robust Huber-LASSO with tuning constant c = 1.345 (blue), c = 1.20 (light blue), c = 1.80 (dark blue); robust quantile-LASSO (red). Probability values from two-sided paired t-tests were calculated using the standard LASSO as reference.

Supplementary Figure 2.II (Extension of **Figure 2**): Boxplots of the standard squared correlation between the observed and the predicted DEFB119 protein residuals based on standard LASSO (black) and robust Huber-LASSO (blue) for different outlier proportions (0.2% and 1.0%) with simulated protein residuals for individuals with average (**A**) or divergent (**B**) genotypes. Probability values from two-sided paired t-tests are shown in the upper part of each panel.

Supplementary Figure 2.III (Extension of **Figure 2**): Boxplots of Kendall's (left), Spearman's (middle) and Pearson's (right) squared correlation between the observed and the predicted DEFB119 protein residuals based on standard LASSO (black) and robust Huber-LASSO (blue) with simulated protein residuals for individuals with average (**A**) or divergent (**B**) genotypes. Probability values from two-sided paired t-tests are shown in the upper part of each panel.

Supplementary Table 1.I (Extension of **Table 1**): Regularization parameter λ, Jaccard index, and false-positive rate considering other values of the tuning constant for the Huber loss function (c = 1.20 and c = 1.80) and the quantile loss function.

Supplementary Table 1.II (Extension of **Table 1**): Regularization parameter λ, Jaccard index, and false-positive rate for different outlier proportions (0.2% and 1.0%).

Supplementary Table 2.I (Extension of **Table 2**): Explained variance, minor allele frequency (MAF), and reported effect size of the three SNPs associated with the plasma levels of protein DEFB119, as well as the corresponding true-positive rate and the estimated regression coefficients considering other values of the tuning constant for the Huber loss function (c = 1.20 and c = 1.80) and the quantile loss function.

Supplementary Table 2.II (Extension of **Table 2**): Explained variance, minor allele frequency (MAF), and reported effect size of the three SNPs associated with the plasma levels of protein DEFB119, as well as the corresponding true-positive rate and the estimated regression coefficients for different outlier proportions (0.2% and 1%).

Supplementary Table 3 (Extension of **Table 3**): Explained variance and the median squared correlation between observed and predicted levels of the metabolites L-carnitine and glutarylcarnitine, the three genes *AGA*, *SNRNP25*, and *XRRA1*, and the proteins DEFB119 and SLAMF7 considering other values of the tuning constant for the Huber loss function (c = 1.20 and c = 1.80) and the quantile loss function. The correlation coefficient for the expression levels of *XRRA1* was -.0629 (standard LASSO) and .0129 (Robust Huber-LASSO c = 1.345).

Supplementary Source Code

Supplementary Figure 1.I (Extension of **Figure 1**): (**A**) Dependence of the regularization parameter λ (left panel) and of the estimated regression coefficient for the SNP most strongly associated with the plasma level of protein DEFB119 (right panel) on the value assigned to the residual for the individual with the average genotype considering the quantile loss function (**B**) Dependence of the regularization parameter λ (left panel) and dependence of the estimated regression coefficient for the SNP most strongly associated with the plasma level of protein DEFB119 (right panel) on the value assigned to the residual for the individual with the divergent genotype considering the quantile loss function.

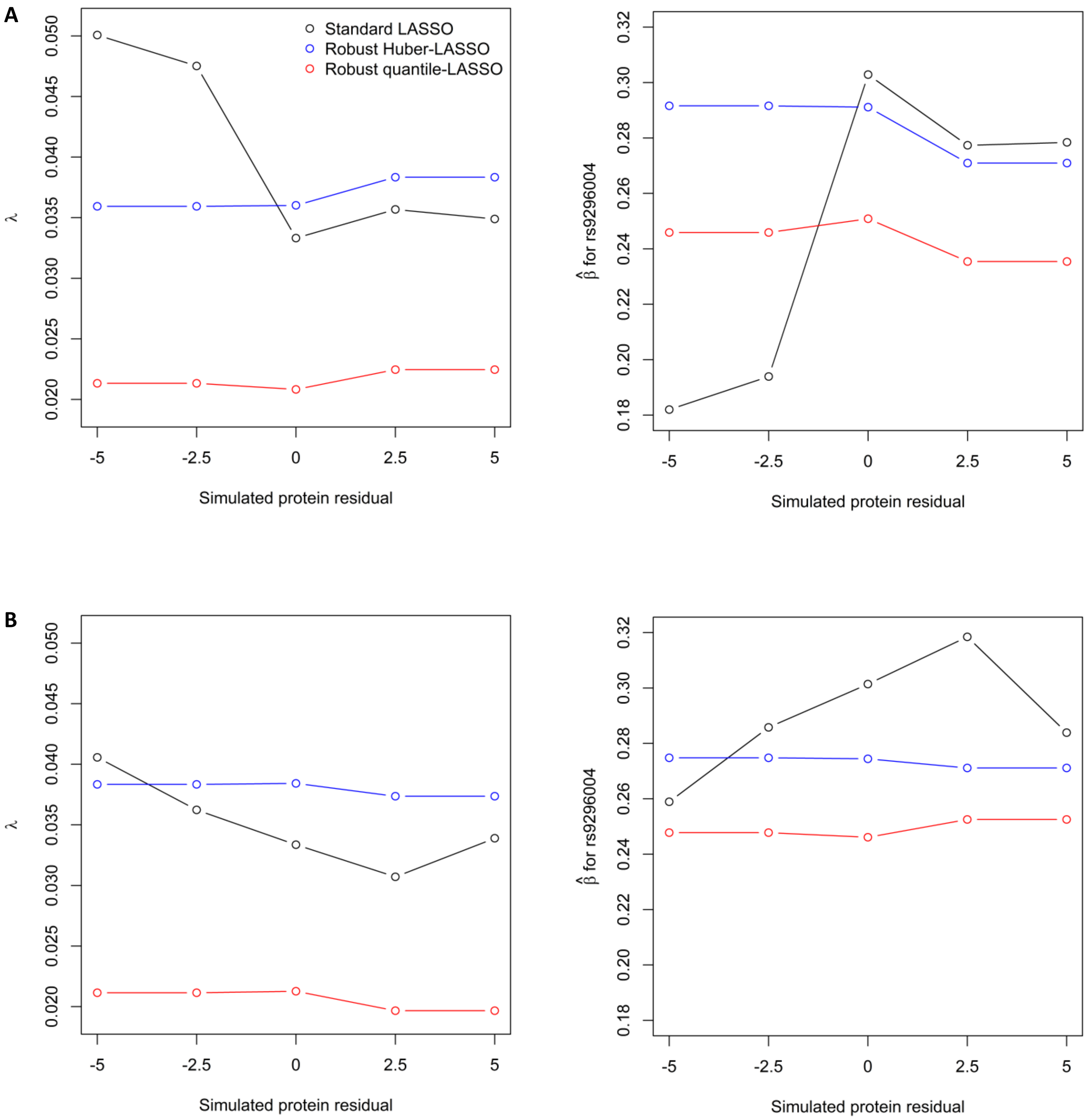

Supplementary Figure 1.II (Extension of **Figure 1**): Dependence of the ratios between the estimated regression coefficients for the three SNPs most strongly associated with the plasma level of protein DEFB119 on the value assigned to the residual for the individual with average (**A**) and divergent (**B**) genotype.

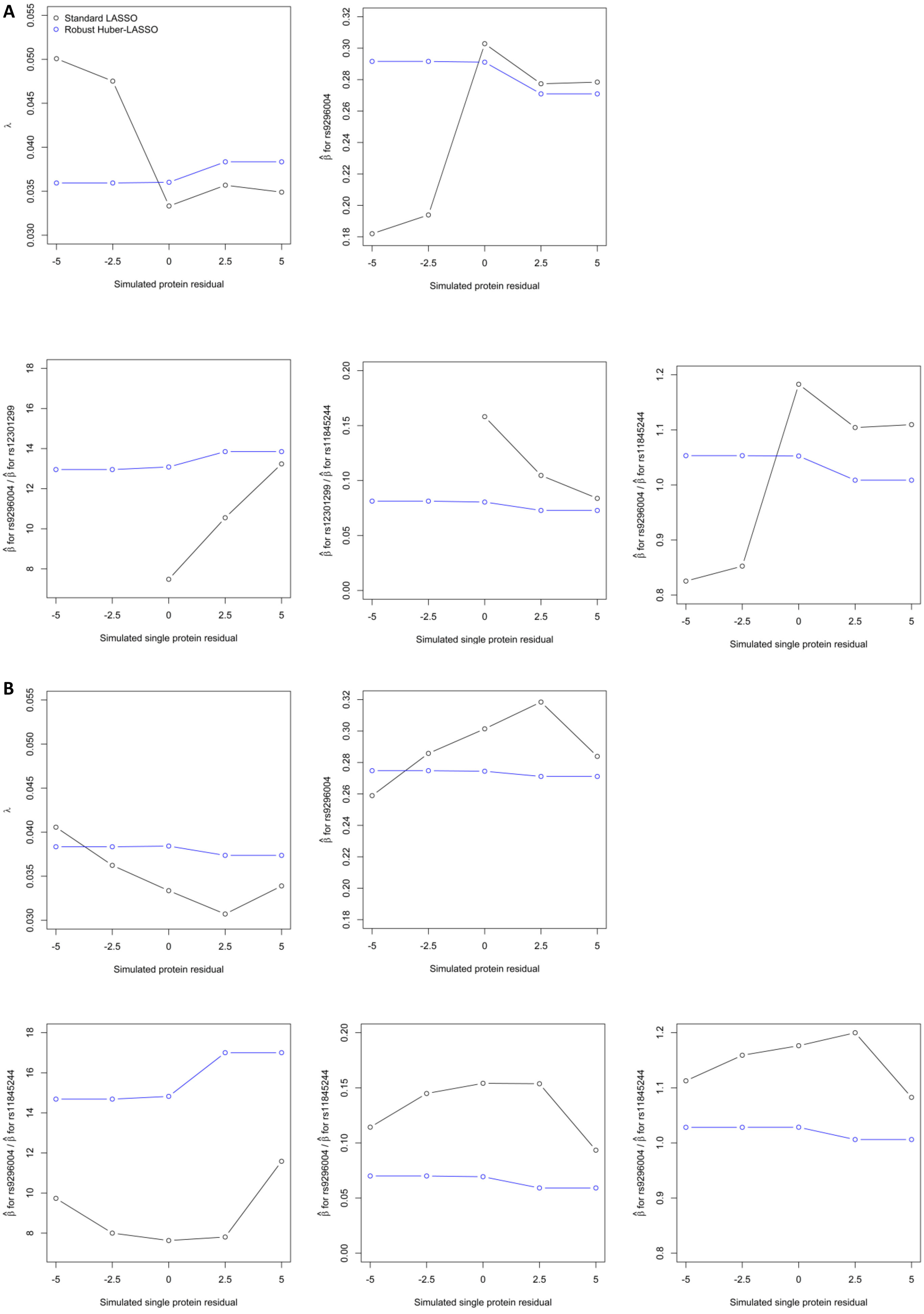

Supplementary Figure 2.I (Extension of **Figure 2**): Boxplots of the standard squared correlation between the observed and the predicted DEFB119 protein residuals considering other values of the tuning constant for the Huber loss function (*c* = 1.20 and *c* = 1.80) and the quantile loss function with simulated protein residuals for individuals with average (**A**) or divergent (**B**) genotypes. From left to right: Standard LASSO (black), robust Huber-LASSO with tuning constant *c* = 1.345 (blue), *c* = 1.20 (light blue), *c* = 1.80 (dark blue), robust quantile-LASSO (red). Probability values from two-sided paired t-tests were calculated using the standard LASSO as reference.

A **B**

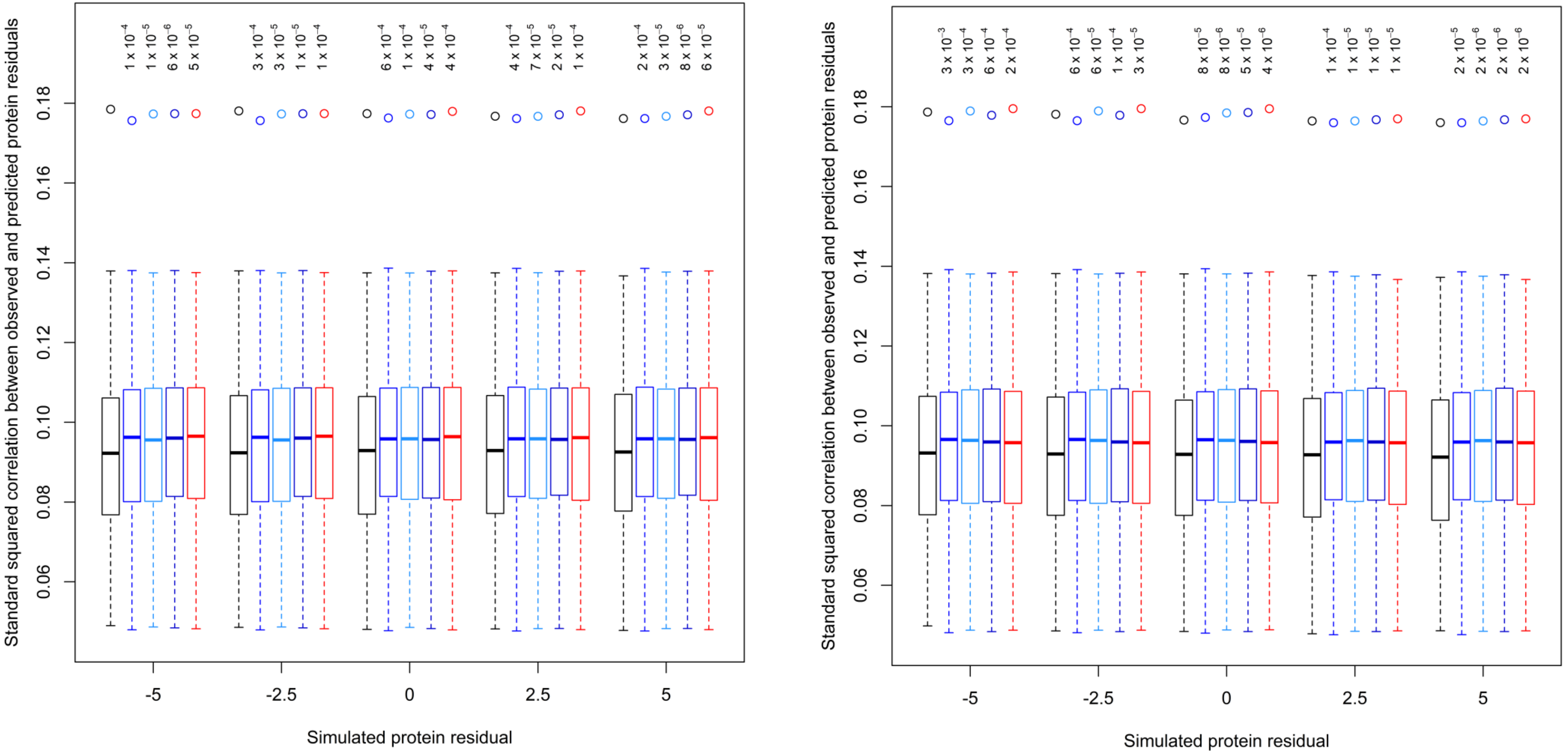

Supplementary Figure 2.II (Extension of **Figure 2**): Boxplots of the standard squared correlation between the observed and the predicted DEFB119 protein residuals based on standard LASSO (black) and robust Huber-LASSO (blue) for different outlier proportions (0.2% and 1.0%) with simulated protein residuals for individuals with average (**A**) or divergent (**B**) genotypes. Probability values from two-sided paired t-tests are shown in the upper part of the figures.

A **B**

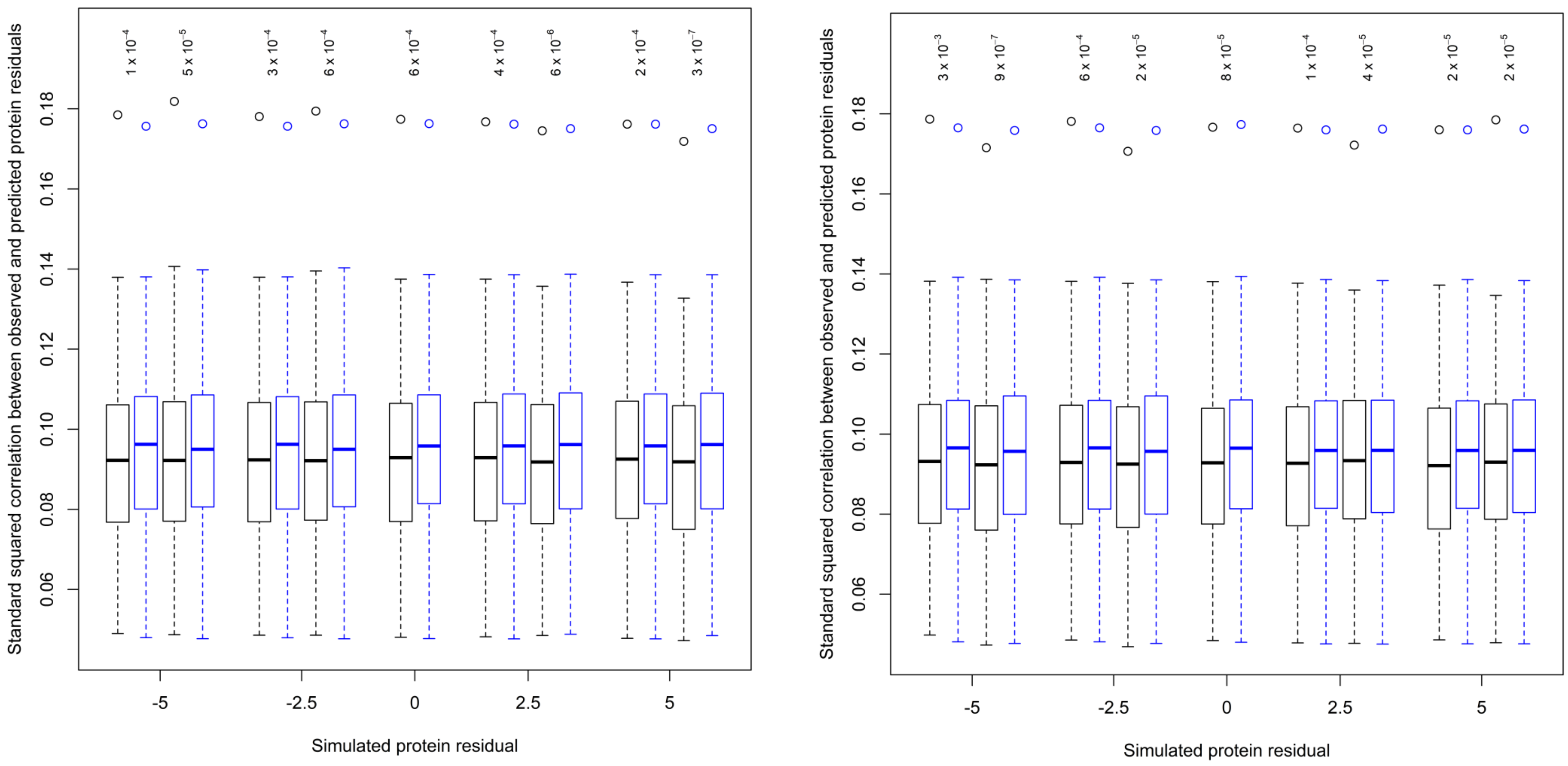

Supplementary Figure 2.III (Extension of **Figure 2**): Boxplots of Kendall's (left), Spearman's (middle) and Pearson's (right) squared correlation between the observed and the predicted DEFB119 protein residuals based on standard LASSO (black) and robust Huber-LASSO (blue) with simulated protein residuals for individuals with average (**A**) or divergent (**B**) genotypes. Probability values from two-sided paired t-tests are shown in the upper part of the figures.

A

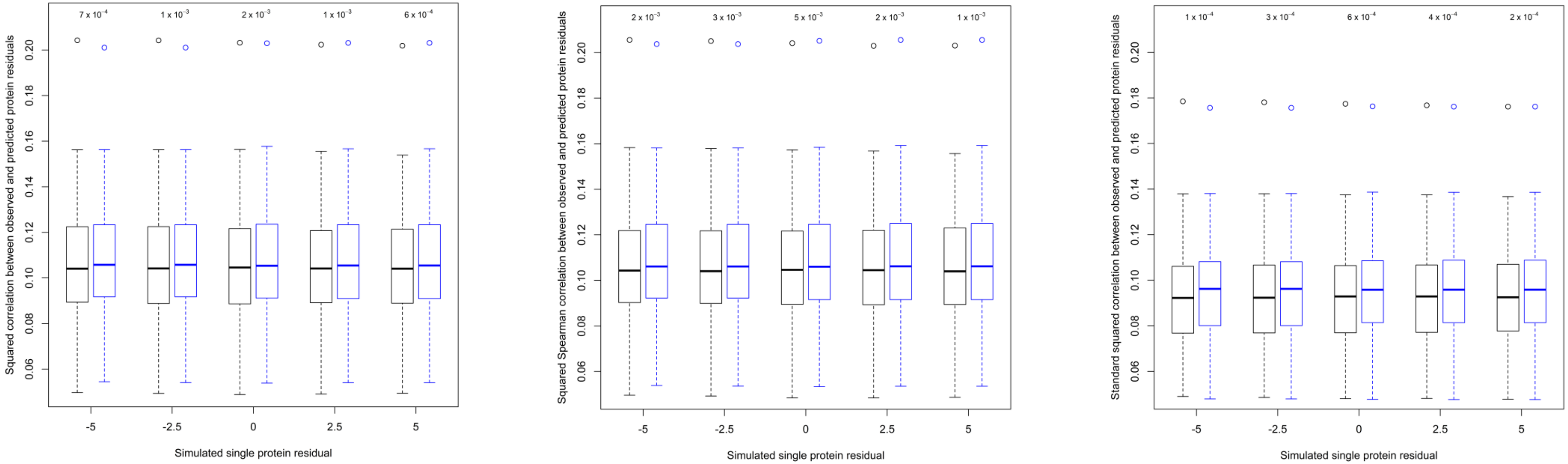

B

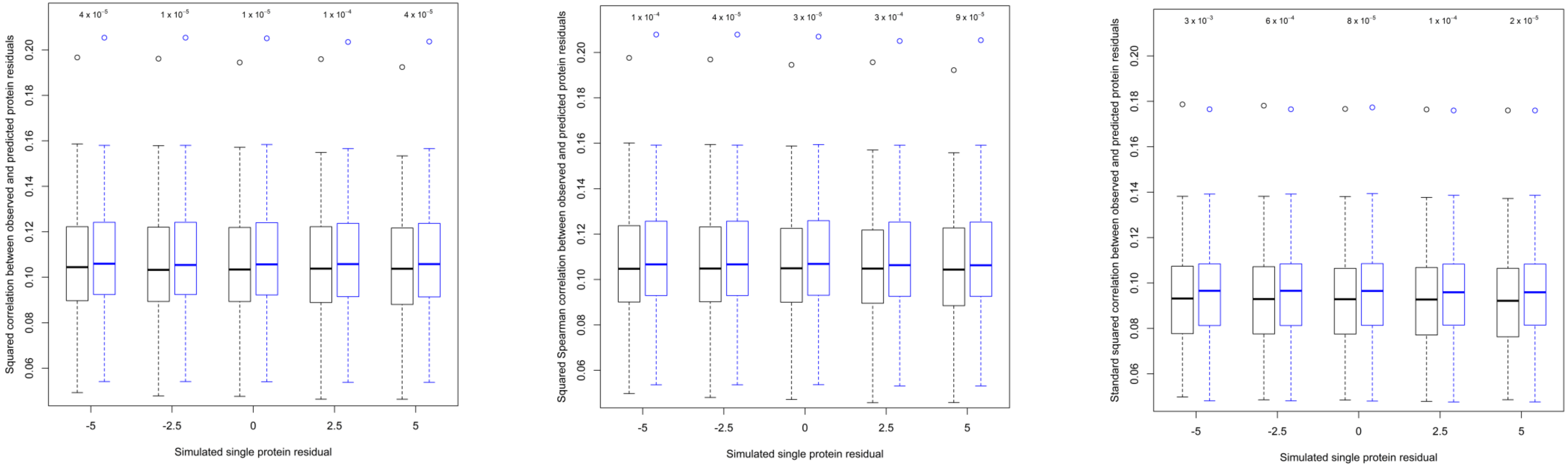

Supplementary Table 1.I (Extension of **Table 1**): Regularization parameter λ, Jaccard index, and false-positive rate considering other values of the tuning constant for the Huber loss function (*c* = 1.20 and *c* = 1.80) and the quantile loss function.

		**Standard LASSO**			**Robust Huber-LASSO**									**Robust quantile-LASSO**		
**Simulated**					**c = 1.345**			**c = 1.20**			**c = 1.80**			**τ = 0.50**		
**outlying**		**Median**	**Median**	**Median**	**Median**	**Median**	**Median**	**Median**	**Median**	**Median**	**Median**	**Median**	**Median**	**Median**	**Median**	**Median**
**phenotype**	**Genotype**	**λ**	**Jaccard index**	**false-positive rate**	**λ**	**Jaccard index**	**false-positive rate**	**λ**	**Jaccard index**	**false-positive rate**	**λ**	**Jaccard index**	**false-positive rate**	**λ**	**Jaccard index**	**false-positive rate**
None		0.041	0.125	0.020	0.044	0.111	0.018	0.044	0.108	0.021	0.043	0.111	0.019	0.022	0.111	0.017
5	Low-leverage	0.044	0.115	0.020	0.044	0.109	0.018	0.044	0.108	0.020	0.047	0.111	0.018	0.023	0.111	0.018
-5	(average)	0.043	0.125	0.019	0.046	0.111	0.016	0.045	0.112	0.021	0.040	0.114	0.017	0.022	0.111	0.018
5	High-leverage	0.044	0.119	0.020	0.044	0.111	0.019	0.044	0.108	0.020	0.043	0.114	0.021	0.023	0.109	0.017
-5	(divergent)	0.043	0.123	0.020	0.044	0.111	0.018	0.044	0.109	0.021	0.044	0.111	0.021	0.023	0.107	0.017

Supplementary Table 1.II (Extension of **Table 1**): Regularization parameter λ, Jaccard index, and false-positive rate for different outlier proportions (0.2% and 1.0%).

Simulated outlying phenotype	Genotype	Proportion of outliers (%)	Standard LASSO Median λ	Standard LASSO Median Jaccard index	Standard LASSO Median false-positive rate	Robust Huber-LASSO Median λ	Robust Huber-LASSO Median Jaccard index	Robust Huber-LASSO Median false-positive rate
None			0.041	0.125	0.020	0.044	0.111	0.018
5	Low-leverage (average)	0.2	0.044	0.115	0.020	0.044	0.109	0.018
-5		0.2	0.043	0.125	0.019	0.046	0.111	0.016
5	High-leverage (divergent)	0.2	0.044	0.119	0.020	0.044	0.111	0.019
-5		0.2	0.043	0.123	0.020	0.044	0.111	0.018
5	Low-leverage (average)	1.0	0.051	0.095	0.016	0.044	0.105	0.018
-5		1.0	0.047	0.111	0.020	0.045	0.118	0.016
5	High-leverage (divergent)	1.0	0.049	0.103	0.018	0.044	0.106	0.018
-5		1.0	0.050	0.111	0.016	0.044	0.107	0.020

Supplementary Table 2.I (Extension of **Table 2**): Explained variance, minor allele frequency (MAF), and reported effect size of the three SNPs associated with the plasma levels of protein DEFB119, as well as the corresponding true-positive rate and the estimated regression coefficients considering other values of the tuning constant for the Huber loss function (*c* = 1.20 and *c* = 1.80) and the quantile loss function.

				**Standard LASSO**				**Robust Huber-LASSO**												**Robust quantile-LASSO**			
								**c = 1.345**				**c = 1.20**				**c = 1.80**				**τ = 0.50**			
**Associated SNP**	**Explained variance**	**MAF**	**Reported effect size**	**True-positive rate**	**Estimated coefficient** Mean	95% CI		**True-positive rate**	**Estimated coefficient** Mean	95% CI		**True-positive rate**	**Estimated coefficient** Mean	95% CI		**True-positive rate**	**Estimated coefficient** Mean	95% CI		**True-positive rate**	**Estimated coefficient** Mean	95% CI	
No simulated outlier																							
rs9296004	0.055	0.082	0.606	0.970	0.253	0.233	0.273	0.970	0.281	0.253	0.309	0.950	0.279	0.249	0.308	0.960	0.286	0.257	0.315	0.970	0.278	0.250	0.305
rs11845244	0.027	0.354	0.241	0.850	0.094	0.082	0.106	0.850	0.103	0.087	0.118	0.830	0.102	0.086	0.118	0.850	0.105	0.090	0.121	0.840	0.105	0.089	0.121
rs12301299	0.018	0.163	0.254	0.960	0.127	0.114	0.139	0.840	0.095	0.082	0.109	0.860	0.094	0.080	0.107	0.870	0.096	0.082	0.109	0.870	0.094	0.081	0.108
**Average genotype**																							
Simulated protein residual = 5																							
rs9296004	0.055	0.082	0.606	0.930	0.229	0.208	0.250	0.960	0.279	0.251	0.308	0.950	0.280	0.250	0.309	0.960	0.284	0.255	0.313	0.960	0.275	0.247	0.304
rs11845244	0.027	0.354	0.241	0.790	0.078	0.066	0.091	0.840	0.100	0.085	0.116	0.840	0.100	0.085	0.116	0.860	0.104	0.088	0.119	0.830	0.103	0.087	0.120
rs12301299	0.018	0.163	0.254	0.940	0.122	0.109	0.135	0.830	0.096	0.082	0.110	0.860	0.095	0.082	0.109	0.860	0.096	0.083	0.110	0.870	0.096	0.083	0.110
Simulated protein residual = -5																							
rs9296004	0.055	0.082	0.606	0.950	0.247	0.226	0.269	0.950	0.287	0.258	0.317	0.960	0.283	0.144	0.312	0.970	0.279	0.251	0.307	0.970	0.279	0.251	0.307
rs11845244	0.027	0.354	0.241	0.860	0.095	0.082	0.108	0.860	0.107	0.091	0.123	0.850	0.105	0.089	0.121	0.840	0.102	0.087	0.118	0.820	0.107	0.090	0.124
rs12301299	0.018	0.163	0.254	0.960	0.121	0.108	0.134	0.870	0.096	0.083	0.109	0.870	0.096	0.083	0.110	0.860	0.094	0.080	0.107	0.870	0.096	0.083	0.109
**Divergent genotype**																							
Simulated protein residual = 5																							
rs9296004	0.055	0.082	0.606	0.960	0.230	0.21	0.251	0.960	0.281	0.253	0.310	0.950	0.281	0.251	0.310	0.970	0.283	0.254	0.312	0.940	0.273	0.243	0.303
rs11845244	0.027	0.354	0.241	0.810	0.090	0.078	0.103	0.840	0.104	0.088	0.120	0.840	0.103	0.087	0.119	0.870	0.104	0.089	0.119	0.830	0.098	0.082	0.114
rs12301299	0.018	0.163	0.254	0.950	0.120	0.107	0.133	0.860	0.097	0.084	0.111	0.850	0.097	0.084	0.111	0.870	0.098	0.084	0.111	0.870	0.095	0.081	0.108
Simulated protein residual = -5																							
rs9296004	0.055	0.082	0.606	0.960	0.247	0.225	0.269	0.950	0.288	0.259	0.316	0.950	0.282	0.252	0.311	0.950	0.284	0.255	0.312	0.930	0.278	0.248	0.309
rs11845244	0.027	0.354	0.241	0.800	0.082	0.070	0.095	0.850	0.103	0.088	0.119	0.840	0.100	0.084	0.116	0.860	0.102	0.087	0.117	0.820	0.096	0.080	0.112
rs12301299	0.018	0.163	0.254	0.970	0.126	0.113	0.138	0.880	0.097	0.084	0.110	0.870	0.097	0.083	0.111	0.870	0.098	0.084	0.111	0.860	0.096	0.083	0.110

Supplementary Table 2.II (Extension of **Table 2**): Explained variance, minor allele frequency (MAF), and reported effect size of the three SNPs associated with the plasma levels of protein DEFB119, as well as the corresponding true-positive rate and the estimated regression coefficients for different outlier proportions (0.2% and 1%).

					**Standard LASSO**				**Robust Huber-LASSO**			
**Associated SNP**	**Explained variance**	**MAF**	**Reported effect size**	**Proportion of outliers (%)**	**True-positive rate**	**Estimated coefficient**			**True-positive rate**	**Estimated coefficient**		
						Mean	95% CI			Mean	95% CI	
No simulated outlier												
rs9296004	0.055	0.082	0.606		0.970	0.253	0.233	0.273	0.970	0.281	0.253	0.309
rs11845244	0.027	0.354	0.241		0.850	0.094	0.082	0.106	0.850	0.103	0.087	0.118
rs12301299	0.018	0.163	0.254		0.960	0.127	0.114	0.139	0.840	0.095	0.082	0.109
**Average genotype**												
Simulated protein residual = 5												
rs9296004	0.055	0.082	0.606	0.2	0.930	0.229	0.208	0.250	0.960	0.279	0.251	0.308
rs11845244	0.027	0.354	0.241	0.2	0.790	0.078	0.066	0.091	0.840	0.100	0.085	0.116
rs12301299	0.018	0.163	0.254	0.2	0.940	0.122	0.109	0.135	0.830	0.096	0.082	0.110
Simulated protein residual = -5												
rs9296004	0.055	0.082	0.606	0.2	0.950	0.247	0.226	0.269	0.950	0.287	0.258	0.317
rs11845244	0.027	0.354	0.241	0.2	0.860	0.095	0.082	0.108	0.860	0.107	0.091	0.123
rs12301299	0.018	0.163	0.254	0.2	0.960	0.121	0.108	0.134	0.870	0.096	0.083	0.109
**Divergent genotype**												
Simulated protein residual = 5												
rs9296004	0.055	0.082	0.606	0.2	0.960	0.230	0.21	0.251	0.960	0.281	0.253	0.310
rs11845244	0.027	0.354	0.241	0.2	0.810	0.090	0.078	0.103	0.840	0.104	0.088	0.120
rs12301299	0.018	0.163	0.254	0.2	0.950	0.120	0.107	0.133	0.860	0.097	0.084	0.111
Simulated protein residual = -5												
rs9296004	0.055	0.082	0.606	0.2	0.960	0.247	0.225	0.269	0.950	0.288	0.259	0.316
rs11845244	0.027	0.354	0.241	0.2	0.800	0.082	0.070	0.095	0.850	0.103	0.088	0.119
rs12301299	0.018	0.163	0.254	0.2	0.970	0.126	0.113	0.138	0.880	0.097	0.084	0.110
**Average genotype**												
Simulated protein residual = 5												
rs9296004				1.0	0.810	0.161	0.139	0.184	0.950	0.274	0.245	0.304
rs11845244				1.0	0.550	0.049	0.037	0.061	0.770	0.096	0.080	0.112
rs12301299				1.0	0.850	0.110	0.096	0.123	0.830	0.095	0.081	0.108
Simulated protein residual = -5												
rs9296004				1.0	0.890	0.229	0.205	0.253	0.970	0.286	0.257	0.315
rs11845244				1.0	0.810	0.098	0.084	0.112	0.880	0.107	0.091	0.122
rs12301299				1.0	0.860	0.100	0.086	0.115	0.870	0.092	0.078	0.105
**Divergent genotype**												
Simulated protein residual = 5												
rs9296004				1.0	0.850	0.180	0.157	0.203	0.940	0.276	0.246	0.306
rs11845244				1.0	0.720	0.072	0.058	0.086	0.800	0.104	0.087	0.120
rs12301299				1.0	0.870	0.099	0.085	0.113	0.850	0.095	0.081	0.109
Simulated protein residual = -5												
rs9296004				1.0	0.950	0.212	0.187	0.237	0.930	0.290	0.259	0.321
rs11845244				1.0	0.640	0.063	0.05	0.076	0.810	0.101	0.085	0.118
rs12301299				1.0	0.910	0.117	0.103	0.131	0.850	0.100	0.087	0.114

Supplementary Table 3 (Extension of **Table 3**): Explained variance and the median squared correlation between observed and predicted levels of the metabolites L-carnitine and glutarylcarnitine, the three genes *AGA*, *SNRNP25*, and *XRRA1*, and the proteins DEFB119 and SLAMF7 considering other values of the tuning constant for the Huber loss function (*c* = 1.20 and *c* = 1.80) and the quantile loss function. The correlation coefficient for the expression levels of *XRRA1* was -.0629 (standard LASSO) and .0129 (robust Huber-LASSO *c* = 1.345).

		**Median squared correlation**				
	**Explained**	Standard LASSO	Robust Huber-LASSO			Robust quantile-LASSO
**Outcome**	**variance**		c = 1.345	c = 1.20	c = 1.80	τ = 0.50
Glutarylcarnitine metabolite level	0.1350	0.0627	0.0667	0.0667	0.0666	0.0660
L-Carnitine metabolite level	0.1490	0.0399	0.0473	0.0473	0.0479	0.0458
AGA expression level	0.0605	0.0481	0.0524	0.0525	0.0521	0.0517
SNRNP25 expression level	0.0695	0.0454	0.0536	0.0533	0.0538	0.0532
XRRA1 expression level	0.0004	0.0040	0.0001	0.0002	0.0001	0.0002
DEFB119 protein level	0.0993	0.0919	0.0942	0.0955	0.0941	0.0941
SLAMF7 protein level	0.1084	0.1038	0.1073	0.1073	0.1081	0.1073

Supplementary source code

This section provides

1. Source code for the simulation of outlying protein levels for individuals with average genotypes (low-leverage observations) including the calculation of the statistical parameters
2. Source code for the real data applications

Note: The working directories have to be set accordingly in each R script. Be aware of time consuming computations.

Simulation study

Source code for the simulation of outlying protein levels for individuals with average genotypes (low-leverage observations) comprising the use of standard and robust Huber-LASSO and the calculation of the Jaccard index, false-positive rate, true-positive rate and estimated regression coefficients

Structure:

1. Data preparation
2. Standard and robust Huber-LASSO
3. Calculation of the statistical parameters
   a. Jaccard index
   b. False-positive rate
   c. True-positive rate
   d. Estimated regression coefficients
4. Diagnostic plots

```
# ------------------------------------------------------------------------------------------ #
# ------------------------------------------------------------------------------------------ #
# 1. Data preparation
# ------------------------------------------------------------------------------------------ #
# ------------------------------------------------------------------------------------------ #

#Load packages
library(hqreg)
library(depth)

#Working directory
dir.read <- "Directoy to read data"
dir.res <- "Directory to save results"

#Read genotype data####
setwd(dir.read)
#Genotype data of associated SNPs
X_geno_ass <- read.table("dat_ass.txt", header = T)
#Genotype data of non-associated SNPs
X_geno_add <- read.table("dat_nonass.txt", header = T)
#Combine genotype data
identical(X_geno_ass[,1],X_geno_add[,1])#compare Sample_Names
X <- as.matrix(cbind(X_geno_ass, X_geno_add))
X <- X[,-grep("Sample_Name",colnames(X))]

#Read proteomics data
Proteomics.data <- read.delim("Proteomics data",header = TRUE)

#Compute trivariate depth of individuals
forpca <- Proteomics.data [, c("Sample_Name", "PC1", "PC2", "PC3")]
for (i in 1:length(forpca$Sample_Name)) {
  forpca$depth[i] <- depth(forpca[i, 2:4], forpca[, 2:4], approx = TRUE)
}

#Read DEFB119 values
identical(Proteomics.data$Sample_Name, X[,1])
y <- Proteomics.data$DEFB119.13455.10.3

#Number of iterations
niter <- 100

#Number of individuals per iteration
nind <- 500

#Simulated outlier values
outlier <- c(-5, -2.5, 0, 2.5, 5)

#Define variables for r and r^2 for standard and robust Huber-LASSO
r_standard <- matrix(nrow = niter, ncol = length(outlier))
r2_standard <- matrix(nrow = niter, ncol = length(outlier))
```

```
r_robust <- matrix(nrow = niter, ncol = length(outlier))
r2_robust <- matrix(nrow = niter, ncol = length(outlier))


# ------------------------------------------------------------------------------------- #
# ------------------------------------------------------------------------------------- #
# 2. LASSO and 5-fold outcome prediction
# ------------------------------------------------------------------------------------- #
# ------------------------------------------------------------------------------------- #

for (i in 1:niter) {
  print(paste0("Iteration number ",i," out of ",niter))
  #Set seed
  set.seed(i)
  #Draw samples (for subset definition)
  rd_smp <- sample(1:nrow(X), nind)

  #Define subsets
  fold1 <- rd_smp[1:(round(0.2 * nind, 0))]
  fold2 <- rd_smp[(round(0.2 * nind, 0) + 1):(round(0.4 * nind, 0))]
  fold3 <- rd_smp[(round(0.4 * nind, 0) + 1):(round(0.6 * nind, 0))]
  fold4 <- rd_smp[(round(0.6 * nind, 0) + 1):(round(0.8 * nind, 0))]
  fold5 <- rd_smp[(round(0.8 * nind, 0) + 1):nind]

  for (j in 1:length(outlier)) {
    #LASSO####
    #Select low-leverage observation by highest trivariate depth
    #Note: replace min() by max() to select high-leverage observation
    idx <- which(forpca_rd$depth == min(forpca_rd$depth))[1]
    #Define simulated outlying protein residual
    y_sim <- y[rd_smp]
    y_sim[idx] <- outlier[j]
    X_sim <- X[rd_smp,]

    #Model trained on all individuals
    cv_standard <- cv.hqreg(X_sim,y_sim,method="ls",FUN="hqreg_raw")
    cv_robust <- cv.hqreg(X_sim,y_sim,method="huber",FUN="hqreg_raw")

    #Standard LASSO results
    #Regression parameter
    lambda_standard[(j-1)*niter+i] <- cv_standard$lambda.min
    #Selected SNPs
    snps_standard[
      (j-1)*niter+i, 1:(predict(cv_standard, lambda=cv_standard$lambda.min, type="nvars")+1)
      ] <- names(coef(cv_standard, cv_standard$lambda.min)[
              coef(cv_standard,cv_standard$lambda.min)!=0])
    #Regression coefficient estimates of selected SNPs
    coef_standard[
      (j-1)*niter+i, 1:(predict(cv_standard, lambda=cv_standard$lambda.min, type="nvars")+1)
      ] <- coef(cv_standard, cv_standard$lambda.min)[
              coef(cv_standard, cv_standard$lambda.min)!=0]

    #Robust Huber-LASSO results
    #Regression parameter
    lambda_robust[(j-1)*niter+i] <- cv_robust$lambda.min
    #Selected SNPs
    snps_robust[
      (j-1)*niter+i, 1:(predict(cv_robust, lambda=cv_robust$lambda.min, type="nvars")+1)
      ] <- names(coef(cv_robust, cv_robust$lambda.min)[
              coef(cv_robust, cv_robust$lambda.min)!=0])
    #Regression coefficient estimates of selected SNPs
    coef_robust[
      (j-1)*niter+i, 1:(predict(cv_robust, lambda=cv_robust$lambda.min, type="nvars")+1)
      ] <- coef(cv_robust, cv_robust$lambda.min)[coef(cv_robust, cv_robust$lambda.min)!=0]


    #5-fold prediction####
    #Select low-leverage observation in Fold2-5
    #Note: replace min() by max() to define high-leverage observation
    idx <- which(which(forpca$depth == min(forpca$depth[c(fold2, fold3, fold4, fold5)])) %in%
                   c(fold2, fold3, fold4, fold5))[1]
    #Define simulated outlying protein residual
    y_sim <- y
    y_sim[idx] <- outlier[j]

    #Model trained on Fold2-5
    cv_standard <-
      cv.hqreg(X[-fold1, ], y_sim[-fold1], method = "ls", FUN = "hqreg_raw", seed = 111)
    cv_robust <-
      cv.hqreg(X[-fold1, ], y_sim[-fold1], method = "huber", FUN = "hqreg_raw", seed = 111)


    #Plasma protein residuals prediction for Fold1
    pred_standard[1:(round(0.2 * nind, 0))] <-
      predict(cv_standard, X[fold1, ], lambda = "lambda.min")
    pred_robust[1:(round(0.2 * nind, 0))] <-
      predict(cv_robust, X[fold1, ], lambda = "lambda.min")

    #Select low-leverage observation in Fold1 + Fold3-5
    #Note: replace min() by max() to define high-leverage observation
    idx <- which(which(forpca$depth == min(forpca$depth[c(fold1, fold3, fold4, fold5)])) %in%
                   c(fold1, fold3, fold4, fold5))[1]
    #Define simulated outlying protein residual
    y_sim <- y
    y_sim[idx] <- outlier[j]
```

```
    #Model trained on Fold1 + Fold3-5
    cv_standard <-
      cv.hqreg(X[-fold2, ], y_sim[-fold2], method = "ls", FUN = "hqreg_raw", seed = 111)
    cv_robust <-
      cv.hqreg(X[-fold2, ], y_sim[-fold2], method = "huber", FUN = "hqreg_raw", seed = 111)

    #Predict plasma protein residual for Fold2
    pred_standard[(round(0.2 * nind, 0) + 1):(round(0.4 * nind, 0))] <-
      predict(cv_standard, X[fold2, ], lambda = "lambda.min")
    pred_robust[(round(0.2 * nind, 0) + 1):(round(0.4 * nind, 0))] <-
      predict(cv_robust, X[fold2, ], lambda = "lambda.min")

    #Select low-leverage observation in Fold1-2 + Fold4-5
    #Note: replace min() by max() to define high-leverage observation
    idx <- which(which(forpca$depth == min(forpca$depth[c(fold1, fold2, fold4, fold5)])) %in%
                   c(fold1, fold2, fold4, fold5))[1]
    #Define simulated outlying protein residual
    y_sim <- y
    y_sim[idx] <- outlier[j]

    #Model trained on Fold1-2 + Fold4-5
    cv_standard <-
      cv.hqreg(X[-fold3, ], y_sim[-fold3], method = "ls", FUN = "hqreg_raw", seed = 111)
    cv_robust <-
      cv.hqreg(X[-fold3, ], y_sim[-fold3], method = "huber", FUN = "hqreg_raw", seed = 111)

    #Predict plasma protein residual for Fold3
    pred_standard[(round(0.4 * nind, 0) + 1):(round(0.6 * nind, 0))] <-
      predict(cv_standard, X[fold3, ], lambda = "lambda.min")
    pred_robust[(round(0.4 * nind, 0) + 1):(round(0.6 * nind, 0))] <-
      predict(cv_robust, X[fold3, ], lambda = "lambda.min")

    #Select low-leverage observation in Fold1-3 + Fold5
    #Note: replace min() by max() to define high-leverage observation
    idx <- which(which(forpca$depth == min(forpca$depth[c(fold1, fold2, fold3, fold5)])) %in%
                   c(fold1, fold2, fold3, fold5))[1]
    #Define simulated outlying protein residual
    y_sim <- y
    y_sim[idx] <- outlier[j]

    #Model trained on Fold1-3 + Fold5
    cv_standard <-
      cv.hqreg(X[-fold4, ], y_sim[-fold4], method = "ls", FUN = "hqreg_raw", seed = 111)
    cv_robust <-
      cv.hqreg(X[-fold4, ], y_sim[-fold4], method = "huber", FUN = "hqreg_raw", seed = 111)

    #predict plasma protein residual for Fold4
    pred_standard[(round(0.6 * nind, 0) + 1):(round(0.8 * nind, 0))] <-
      predict(cv_standard, X[fold4, ], lambda = "lambda.min")
    pred_robust[(round(0.6 * nind, 0) + 1):(round(0.8 * nind, 0))] <-
      predict(cv_robust, X[fold4, ], lambda = "lambda.min")

    #Select low-leverage observation in Fold1-4
    #Note: replace min() by max() to define high-leverage observation
    idx <- which(which(forpca$depth == min(forpca$depth[c(fold1, fold2, fold3, fold4)])) %in%
                   c(fold1, fold2, fold3, fold4))[1]
    #Define simulated outlying protein residual
    y_sim <- y
    y_sim[idx] <- outlier[j]

    #Model trained on Fold1-4
    cv_standard <-
      cv.hqreg(X[-fold5, ], y_sim[-fold5], method = "ls", FUN = "hqreg_raw", seed = 111)
    cv_robust <-
      cv.hqreg(X[-fold5, ], y_sim[-fold5], method = "huber", FUN = "hqreg_raw", seed = 111)

    #Predict plasma protein residual for Fold5
    pred_standard[(round(0.8 * nind, 0) + 1):nind] <-
      predict(cv_standard, X[fold5, ], lambda = "lambda.min")
    pred_robust[(round(0.8 * nind, 0) + 1):nind] <-
      predict(cv_robust, X[fold5, ], lambda = "lambda.min")

    #Reorder protein residuals to calculate the correlation
    y_true <- y[rd_smp]

    #Correlation of observed and predicted plasma protein residuals
    r_standard[i, j] <- cor(y_true, pred_standard,method="kendall")
    r_robust[i, j] <- cor(y_true, pred_robust,method="kendall")

  }
}


# ------------------------------------------------------------------------------------- #
# ------------------------------------------------------------------------------------- #
# 3. Statistical parameters
# ------------------------------------------------------------------------------------- #
# ------------------------------------------------------------------------------------- #

#a. Jaccard index####
#Initiate variables for standard and robust Jaccard index
jaccard_standard_raw <- matrix(nrow = niter * length(outlier), ncol = (niter - 1))
jaccard_robust_raw <- matrix(nrow = niter * length(outlier), ncol = (niter - 1))

jaccard_standard <- matrix(nrow = (niter * (niter - 1)) / 2, ncol = length(outlier))
```

```
jaccard_robust <- matrix(nrow = (niter * (niter - 1)) / 2, ncol = length(outlier))

for (m in 1:length(outlier)) {
  for (i in ((1 * (m - 1) * niter) + 1):((niter * m) - 1)) {
    for (j in (i + 1):(niter * m)) {
      #Compute Jaccard index from standard LASSO
      jaccard_standard_raw[i, j - 1 - ((m - 1) * niter)] <-
        length(intersect(snps_standard[i, which(!(is.na(snps_standard[i, ])))[-1]],
                         snps_standard[j, which(!(is.na(snps_standard[j, ])))[-1]])) /
        length(union(snps_standard[i, which(!(is.na(snps_standard[i, ])))[-1]],
                     snps_standard[j, which(!(is.na(snps_standard[j, ])))[-1]]))
      if (is.na(jaccard_standard_raw[i, j - 1 - ((m - 1) * niter)])) {
        jaccard_standard_raw[i, j - 1 - ((m - 1) * niter)] <- 1
      }
      #Compute Jaccard index from robust Huber-LASSO
      jaccard_robust_raw[i, j - 1 - ((m - 1) * niter)] <-
        length(intersect(snps_robust[i, which(!(is.na(snps_robust[i, ])))[-1]],
                         snps_robust[j, which(!(is.na(snps_robust[j, ])))[-1]])) /
        length(union(snps_robust[i, which(!(is.na(snps_robust[i, ])))[-1]],
                     snps_robust[j, which(!(is.na(snps_robust[j, ])))[-1]]))
      if (is.na(jaccard_robust_raw[i, j - 1 - ((m - 1) * niter)])) {
        jaccard_robust_raw[i, j - 1 - ((m - 1) * niter)] <- 1
      }
    }
  }
  #Order Jaccard index by simulated outlying protein residual
  jaccard_standard[1:length(which(!(is.na(
    as.numeric(jaccard_standard_raw[(1 + (niter * (m - 1))):(niter * m),]))))), m] <-
    as.numeric(jaccard_standard_raw[(1 + (niter * (m - 1))):(niter * m),])[which(!(is.na(
      as.numeric(jaccard_standard_raw[(1 + (niter * (m - 1))):(niter * m),]))))]
  jaccard_robust[1:length(which(!(is.na(
    as.numeric(jaccard_robust_raw[(1 + (niter * (m - 1))):(niter * m), ]))))), m] <-
    as.numeric(jaccard_robust_raw[(1 + (niter * (m - 1))):(niter * m), ])[which(!(is.na(
      as.numeric(jaccard_robust_raw[(1 + (niter * (m - 1))):(niter * m), ]))))]
}


#b. False-positive rate####
#Non-associated SNP IDs
snps_nonass <- colnames(X_geno_add[,-grep("Sample_Name",colnames(X_geno_add))])

#Initiate variables for count of selected non-associated SNPs
count_standard <- matrix(nrow = niter, ncol = length(snps_nonass))
count_robust <- matrix(nrow = niter, ncol = length(snps_nonass))

#Initiate variables for standard and robust false-positive rate
type1_standard <- matrix(nrow = niter, ncol = length(outlier))
type1_robust <- matrix(nrow = niter, ncol = length(outlier))

for (m in 1:length(outlier)) {
  snps_standard_subset <- snps_standard[(1 + (niter * (m - 1))):(m * niter), ]
  coef_standard_subset <- coef_standard[(1 + (niter * (m - 1))):(m * niter), ]

  snps_robust_subset <- snps_robust[(1 + (niter * (m - 1))):(m * niter), ]
  coef_robust_subset <- coef_robust[(1 + (niter * (m - 1))):(m * niter), ]

  for (i in 1:niter) {
    for (j in 1:length(snps_nonass)) {
      count_standard[i, j] <-
        length(which(snps_standard_subset[i, ] %in% snps_nonass[j]))
      count_robust[i, j] <-
        length(which(snps_robust_subset[i, ] %in% snps_nonass[j]))
    }

    type1_standard[i, m] <- sum(count_standard[i, ]) / length(snps_nonass)
    type1_robust[i, m] <- sum(count_robust[i, ]) / length(snps_nonass)
  }
}

#c. True-positive rate####
#Associated SNP IDs
snps_ass <- colnames(X_geno_ass)[,-grep("Sample_Name",colnames(X_geno_ass))]

#Initiate variables for count of selected associated SNPs
count_standard <- numeric(niter)
count_robust <- numeric(niter)

#Initiate variables for standard and robust true-positive rate
power_standard <- matrix(nrow = length(snps_ass), ncol = length(outlier))
power_robust <- matrix(nrow = length(snps_ass), ncol = length(outlier))

for (i in 1:length(snps_ass)) {
  for (j in 1:length(outlier)) {
    #Subset selected SNPs and coefficient estimates by simulated outlying protein residual
    snps_standard_subset <- snps_standard[(1 + (niter * (j - 1))):(j * niter), ]
    coef_standard_subset <- coef_standard[(1 + (niter * (j - 1))):(j * niter), ]

    snps_robust_subset <- snps_robust[(1 + (niter * (j - 1))):(j * niter), ]
    coef_robust_subset <- coef_robust[(1 + (niter * (j - 1))):(j * niter), ]


    for (k in 1:niter) {
      #Check selection of associated SNPs in each iteration
      count_standard[k] <- length(which(snps_standard_subset[k, ] %in% snps_ass[i]))
      count_robust[k] <- length(which(snps_robust_subset[k, ] %in% snps_ass[i]))
    }
```

```
    #Compute true-positive rate
    power_standard[i, j] <- sum(count_standard) / niter
    power_robust[i, j] <- sum(count_robust) / niter
  }
}


#d. Estimated regression coefficients####
#Initiate variables for standard and robust regression coefficient estimates
coef_est_standard <-  matrix(nrow = length(snps_ass), ncol = length(outlier) * niter)
coef_est_robust <-  matrix(nrow = length(snps_ass), ncol = length(outlier) * niter)

#Regression coefficient estimates of associated SNP for each simulated outlying protein #residual and
iteration
for (j in 1:length(outlier)) {
  #Subset selected SNPs and coefficient estimates by simulated outlying protein residual
  snps_standard_subset <- snps_standard[(1 + (niter * (j - 1))):(j * niter), ]
  coef_standard_subset <- coef_standard[(1 + (niter * (j - 1))):(j * niter), ]

  snps_robust_subset <- snps_robust[(1 + (niter * (j - 1))):(j * niter), ]
  coef_robust_subset <- coef_robust[(1 + (niter * (j - 1))):(j * niter), ]

  for (i in 1:length(snps_ass)) {
    snps_idx_standard <- which(snps_standard_subset == snps_ass[i])
    if (length(snps_idx_standard) != 0) {
      coef_est_standard[i, (1 + (niter * (j - 1))):((niter * (j - 1)) +
        length(as.vector(coef_standard_subset)[snps_idx_standard]))] <-
         as.vector(coef_standard_subset)[snps_idx_standard]
    }

    snps_idx_robust <- which(snps_robust_subset == snps_ass[i])
    if (length(snps_idx_robust) != 0) {
      coef_est_robust[i, (1 + (niter * (j - 1))):((niter * (j - 1)) +
        length(as.vector(coef_robust_subset)[snps_idx_robust]))] <-
         as.vector(coef_robust_subset)[snps_idx_robust]
    }
  }
}
coef_est_standard[which(is.na(coef_est_standard))] <- 0
coef_est_robust[which(is.na(coef_est_robust))] <- 0

#e. Squared correlation###

#Fisher-consistent version of correlation
transf <- function(r){sin(0.5*pi*r)}
r_standard_transf <- matrix(nrow=nrow(r_standard),ncol=ncol(r_standard))
r_robust_transf <- matrix(nrow=nrow(r_robust),ncol=ncol(r_robust))
for (i in 1:nrow(r_standard)){
  for (j in 1:ncol(r_standard)){
    r_standard__transf[i,j] <- transf(r_standard[i,j])
    r_robust_transf[i,j] <- transf(r_robust[i,j])
  }}

r2_standard_transf<- matrix(ncol=ncol(r_standard_transf),nrow=nrow(r_standard_transf))
r2_robust_transf<- matrix(ncol=ncol(r_robust_transf),nrow=nrow(r_robust_transf))
for (i in 1:nrow(r_standard)){
  for (j in 1:ncol(r_standard)){
    r2_standard_transf[i,j] <- r_standard_transf[i,j]^2
    r2_robust_transf[i,j] <- r_robust_transf[i,j]^2
  }}



# ------------------------------------------------------------------------------------- #
# ------------------------------------------------------------------------------------- #
# 4. Diagnostic plots
# ------------------------------------------------------------------------------------- #
# ------------------------------------------------------------------------------------- #

#Set working directory
setwd(dir.res)

#Create plot as .tif with resolution equal to 600dpi

#Open graphic device
tiff("Figure2.tiff", height = 6, width = 5.5, units = 'in', compression="lzw", res=1200)


#Plot
boxplot(r2_standard_transf [,1],r2_robust_transf [,1],
        r2_standard_transf [,2],r2_robust_transf [,2],
        r2_standard_transf [,3],r2_robust_transf [,3],
        r2_standard_transf [,4],r2_robust_transf [,4],
        r2_standard_transf [,5],r2_robust_transf [,5],
        boxcol=rep(c("black","blue"),1), medcol=rep(c("black","blue"),1),
        whiskcol=rep(c("black","blue"),1), staplecol=rep(c("black","blue"),1),
        outcol=rep(c("black","blue"),1),
        at=c(1:2,4:5,7:8,10:11,13:14), xaxt="n",
        xlab="Simulated protein residual", ylab=""
)
axis(1,at=c(1.5,4.5,7.5,10.5,13.5),labels=c(-5,-2.5,0,2.5,5))
title(ylab="Squared correlation between observed and predicted protein residuals", line=2.5)

#Close device
dev.off()
```

Real data applications

Source code for the real data applications

Structure

1. Data preparation
2. Standard and robust Huber-LASSO
3. Diagnostic plot

```
# ------------------------------------------------------------------------------------ #
# ------------------------------------------------------------------------------------ #
# 1. Data preparation
# ------------------------------------------------------------------------------------ #
# ------------------------------------------------------------------------------------ #

#Load package
library(hqreg)

#Working directory
dir.read <- "Directoy to read data"
dir.res <- "Directory to save results"

#Read data####
setwd(dir.read)
dat <- read.table("dat.txt", header = T, stringsAsFactors = F)
#data containing associated and randomly sampled non-associated variants with outcome

#Genotypes
X <- as.matrix(dat[, grep("rs", colnames(dat))])

#Observed outcome levels
#Note: Replace <outcome> by metabolite, gene or protein name
y <- dat[,grep("<outcome>",colnames(dat))]

#Number of iterations
niter <- 100

#Define variables for r and r^2 for standard and robust Huber-LASSO
r_standard <- as.numeric(niter)
r2_standard <- as.numeric(niter)

r_robust <- as.numeric(niter)
r2_robust <- as.numeric(niter)


# ------------------------------------------------------------------------------------ #
# ------------------------------------------------------------------------------------ #
# 2. LASSO and 5-fold outcome prediction
# ------------------------------------------------------------------------------------ #
# ------------------------------------------------------------------------------------ #

for (i in 1:niter) {
  print(paste0("Iteration number ",i," out of ",niter))
  #Set seed
  set.seed(i)
  #Draw samples (for subset definition)
  samples <- sample(1:nrow(X), nrow(X))
  #Define subsets
  fold1 <- samples[1:(round(0.2 * nrow(X), 0))]
  fold2 <- samples[(round(0.2 * nrow(X), 0) + 1):(round(0.4 * nrow(X), 0))]
  fold3 <- samples[(round(0.4 * nrow(X), 0) + 1):(round(0.6 * nrow(X), 0))]
  fold4 <- samples[(round(0.6 * nrow(X), 0) + 1):(round(0.8 * nrow(X), 0))]
  fold5 <- samples[(round(0.8 * nrow(X), 0) + 1):nrow(X)]

  #Define variables for standard and robust prediction result
  pred_standard <- numeric(nrow(X))
  pred_robust <- numeric(nrow(X))

  #LASSO and prediction for each subset
  #Fold1
  cv_standard <-
    cv.hqreg(X[-fold1, ], y[-fold1], method = "ls", FUN = "hqreg_raw", seed = 111)
  cv_robust <-
    cv.hqreg(X[-fold1, ], y[-fold1], method = "huber", FUN = "hqreg_raw", seed = 111)

  #Prediction of Fold1
  pred_standard[1:(round(0.2 * nrow(X), 0))] <-
    predict(cv_standard, X[fold1, ], lambda = "lambda.min")
  pred_robust[1:(round(0.2 * nrow(X), 0))] <-
    predict(cv_robust, X[fold1, ], lambda = "lambda.min")

  #Fold2
  cv_standard <-
    cv.hqreg(X[-fold2, ], y[-fold2], method = "ls", FUN = "hqreg_raw", seed = 111)
  cv_robust <-
    cv.hqreg(X[-fold2, ], y[-fold2], method = "huber", FUN = "hqreg_raw", seed = 111)
```

```
#Prediction of Fold2
  pred_standard[(round(0.2 * nrow(X), 0) + 1):(round(0.4 * nrow(X), 0))] <-
    predict(cv_standard, X[fold2, ], lambda = "lambda.min")
  pred_robust[(round(0.2 * nrow(X), 0) + 1):(round(0.4 * nrow(X), 0))] <-
    predict(cv_robust, X[fold2, ], lambda = "lambda.min")

  #Fold3
  cv_standard <-
    cv.hqreg(X[-fold3, ], y[-fold3], method = "ls", FUN = "hqreg_raw", seed = 111)
  cv_robust <-
    cv.hqreg(X[-fold3, ], y[-fold3], method = "huber", FUN = "hqreg_raw", seed = 111)

  #Prediction of Fold3
  pred_standard[(round(0.4 * nrow(X), 0) + 1):(round(0.6 * nrow(X), 0))] <-
    predict(cv_standard, X[fold3, ], lambda = "lambda.min")
  pred_robust[(round(0.4 * nrow(X), 0) + 1):(round(0.6 * nrow(X), 0))] <-
    predict(cv_robust, X[fold3, ], lambda = "lambda.min")

  #Fold4
  cv_standard <-
    cv.hqreg(X[-fold4, ], y[-fold4], method = "ls", FUN = "hqreg_raw", seed = 111)
  cv_robust <-
    cv.hqreg(X[-fold4,], y[-fold4], method = "huber", FUN = "hqreg_raw", seed = 111)

  #Prediction of Fold4
  pred_standard[(round(0.6 * nrow(X), 0) + 1):(round(0.8 * nrow(X), 0))] <-
    predict(cv_standard, X[fold4, ], lambda = "lambda.min")
  pred_robust[(round(0.6 * nrow(X), 0) + 1):(round(0.8 * nrow(X), 0))] <-
    predict(cv_robust, X[fold4, ], lambda = "lambda.min")

  #Fold5
  cv_standard <-
    cv.hqreg(X[-fold5,], y[-fold5], method = "ls", FUN = "hqreg_raw", seed = 111)
  cv_robust <-
    cv.hqreg(X[-fold5,], y[-fold5], method = "huber", FUN = "hqreg_raw", seed = 111)

  #Prediction of Fold5
  pred_standard[(round(0.8 * nrow(X), 0) + 1):nrow(X)] <-
    predict(cv_standard, X[fold5,], lambda = "lambda.min")
  pred_robust[(round(0.8 * nrow(X), 0) + 1):nrow(X)] <-
    predict(cv_robust, X[fold5,], lambda = "lambda.min")

  #Reorder outcome levels (for correlation)
  y_true <- y[samples]

  #Correlation of observed and predicted outcome levels
  r_standard[i] <- cor(y_true, pred_standard, method="kendall")
  r_robust[i] <- cor(y_true, pred_robust, method="kendall")
}

#Fisher-consistent version of correlation
transf <- function(r){sin(0.5*pi*r)}
r_standard_transf <- numeric(length(r_standard))
r_robust_transf <- numeric(length(r_robust))
for (i in 1:length(r_standard)){
    r_standard__transf[i] <- transf(r_standard[i])
    r_robust_transf[i] <- transf(r_robust[i])
  }

#Squared correlation
r2_standard_transf<- numeric(length(r_standard_transf))
r2_robust_transf<- numeric(length(r_robust_transf))
for (i in 1:length(r_standard)){
    r2_standard_transf[i] <- r_standard_transf[i]^2
    r2_robust_transf[i] <- r_robust_transf[i]^2
  }


# ------------------------------------------------------------------------------------- #
# ------------------------------------------------------------------------------------- #
# 3. Diagnostic plots
# ------------------------------------------------------------------------------------- #
# ------------------------------------------------------------------------------------- #

#Set working directory
setwd(dir.res)

#Create plot as .tif with resolution equal to 600dpi

#Open graphic device
tiff("Figure3.tiff", height = 8, width = 7.5, units = 'in', compression="lzw", res=1200)

#Plot
boxplot(r2_standard_transf,r2_robust_transf,
        boxcol=c("black","blue"), medcol=c("black","blue"), whiskcol=c("black","blue"),
        staplecol=c("black","blue"), outcol=c("black","blue"),
        at=c(1:2),
        names=c("Standard LASSO","Robust Huber-LASSO"),
        ylab=""
)
title(ylab="Squared correlation between observed and predicted metabolite levels",
      line=2.5)

#Close device
dev.off()
```
